# Structural and functional characterization of the Pro64Ser leptin mutant: Implications for congenital leptin deficiency

**DOI:** 10.1016/j.bpj.2025.08.026

**Published:** 2025-08-28

**Authors:** Bao Quoc Ngo, Outi Lampela, André H. Juffer

**Affiliations:** 1 Faculty of Biochemistry and Molecular Medicine, University of Oulu, PO Box 5400, 90014 Oulu, Finland; 2 Biocenter Oulu and Faculty of Biochemistry and Molecular Medicine, University of Oulu, PO Box 5400, 90014 Oulu, Finland

## Abstract

Congenital leptin deficiency or dysfunction is a form of monogenic childhood obesity. The disease is primarily caused by mutations in the *LEP* gene, which encodes for the expression of a hormone called leptin. The mutations typically impair leptin synthesis, secretion, or binding to the leptin receptor (LepR). The Pro64Ser mutation in leptin, despite not affecting the protein’s stability or its binding affinity to the LepR, completely abolishes the protein’s ability to mediate intracellular signaling via the LepR. To elucidate the mechanism underlying this signal inhibition and to further understand the mechanism of leptin-mediated LepR signal transduction, we performed extensive molecular dynamics simulations of both the wild-type and mutant (MT) leptins. Our simulations reveal that the Pro64Ser mutation increases the rigidity of AB loop N-terminus and thus prevents the loop’s conformational changes required for interaction with the LepR immunoglobulin-like domain (IgD). Conversely, the CD loop of the MT exhibits increased flexibility compared with the wild-type. This elevated flexibility potentially hinders the protein’s transition into helical structure and subsequent interaction with the IgD. Given that the interactions between leptin and the LepR IgD are crucial for the formation of higher-order leptin-LepR assembly and the following intracellular signal transduction, the observed changes in the MT leptin loop dynamics provide a mechanistic explanation for the signaling defects.

## Significance

Unlike other mutations associated with congenital leptin deficiency or dysfunction, which typically result in defects in leptin synthesis, secretion, or its ability to bind to the leptin receptor (LepR), the Pro64Ser mutation does not adversely affect the protein’s stability or its receptor-binding capability. The present work aims to elucidate the structural impact of the Pro64Ser mutation on leptin and, more importantly, to establish the connection between this structural alteration and the impaired LepR signal transduction. This investigation allows for discovering the molecular details of interactions between leptin and the LepR immunoglobulin-like domain, which drive the formation of a higher-order leptin-LepR assembly and serve as a critical step for activating the LepR and intracellular signal transduction.

## Introduction

Childhood obesity is a major global health challenge that negatively impacts children’s physical, social, and/or emotional well-being. Over the period 1975–2016, the global prevalence of obesity in children and adolescents increased from 0.9% to 7.8% in boys and from 0.7% to 5.6% in girls (1). Particularly, it is projected that by 2030, nearly 40 million of children under 5 years old and 254 million of children between 5 and 19 years old will be classified as overweight or obese (2). The consequences of childhood obesity are detrimental to both individuals and the whole society. A study in the United States revealed that the estimated lifetime medical costs for a 10-year-old child with obesity is approximately $16,310 to $19,350 higher than those for a child with a healthy weight (3,4). Furthermore, the total lifetime medical costs for obesity treatment among the US fifth grade students, who remained obese in adulthood, were estimated to be $25 billion higher than those who maintained a healthy weight throughout their lives (4).

From genetic background analyses, the two main types of childhood obesity include monogenic and polygenic obesity. Monogenic obesity is caused by mutations in a single gene, which usually results in its loss of function or haploinsufficiency (5). By contrast, polygenic obesity is associated with several polymorphic genes and their interactions with other environmental factors (5).

Monogenic childhood obesity is described as uncommon. The mutations of only a few genes that are responsible for the progression of the disease have been described. These include *LEP* (leptin), *LepR* (leptin receptor), *PCSK1* (prohormone convertase 1/3), *POMC* (proopiomelanocortin), *SIM1* (single-minded homolog 1), and *MC4R* (melanocortin 4 receptor) (5). Among all forms of monogenic childhood obesity, the only form that can be causally treated is congenital leptin deficiency or dysfunction. Patients with this disease are characterized by intensive hyperphagia from early childhood and abnormally rapid weight gain after birth despite the normal birth weight (6). In addition, other symptoms including impaired satiety, hyperinsulinemia, hypothalamic hypothyroidism, advanced bone age, and hypogonadotropic hypogonadism were also observed (6). In some cases, the patients were even reported to be increasingly susceptible to bacterial infections due to immunological alterations (7).

**Figure 1:**
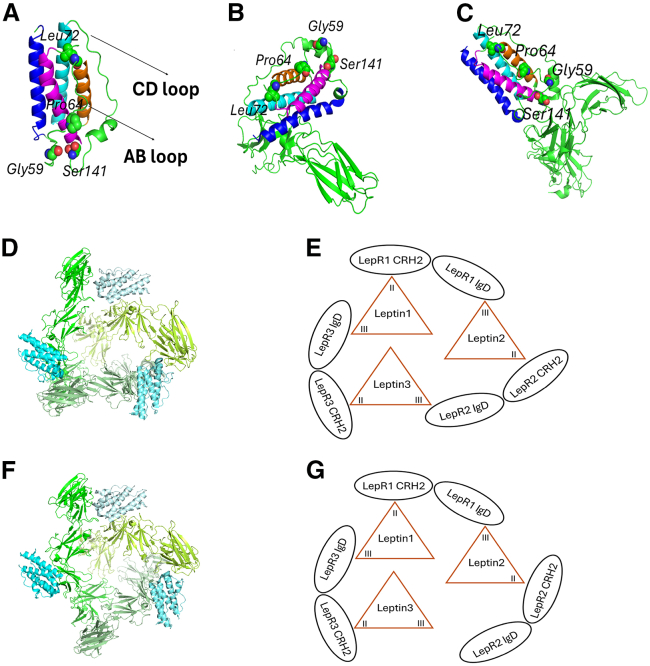
Human leptin structure and leptin-LepR assembly. (*A*) Structure of the modeled human leptin using the crystal structure of human leptin W100E (PDB: 1AX8) (11) as a template. The main four antiparallel α-helices include helix A (from residue Pro23 to His47) in blue, B (from residue Leu72 to Ser88) in orange, C (from residue Arg92 to Lys115) in cyan, and D (from Ser141 to Ser164) in magenta. The WT amino acids Gly59, Pro64, Leu72, and Ser141 are shown as green-colored spheres. (*B*) Interactions between leptin and the LepR CRH2 domain. (*C*) Interactions between leptin and the LepR IgD. In (*B*) and (*C*), the LepR IgD (from residue Val333 to Tyr426) and CRH2 (from residue Ile428 to Val633) domains are colored in forest and lime green, respectively. Leptin α-helices maintain their respective colors as in (*A*). The amino acids Gly59, Pro64, Leu72, and Ser141 are represented as spheres. (*D*) Structure of the closed 3:3 human leptin-LepR complex (PDB: 8AVF) (12). Leptin and the LepR molecules are colored with different shades of cyan and green, respectively. (*E*) Schematic representation of the closed 3:3 complex structure. Leptin engages its binding site II with the LepR CRH2 domain and its binding site III with the IgD. Leptin1, leptin2, and leptin3 correspond to chains A, C, and E, respectively, in the closed 3:3 structure (PDB: 8AVF) (12). Similarly, LepR1, LepR2, and LepR3 correspond to chains B, D, and F. (*F*) Structural representation of the open 3:3 human leptin-LepR complex (PDB: 8AVO) (12). Leptin and the LepR molecules are depicted in different shades of cyan and green, respectively. (*G*) Schematic illustration of the open 3:3 complex. In the open structure, one leptin molecule interacts solely with the LepR CRH2. Conversely, the other two molecules engage with both the CRH2 and IgD domains. Leptin1, leptin2, and leptin3 correspond to the molecules designated as chain (A, C, and E), respectively in the open 3:3 leptin-LepR (PDB: 8AVO) (12). LepR1, LepR2, and LepR3 correspond to chain B, D, and F. The subfigures (A, B, C, D, and F) were generated using PyMOL Molecular Graphics System (open source, Schrödinger, LLC). The amino acid numbering scheme is shown in Figs. S1 and S2. Note that leptin molecule residues are numbered based on the protein’s full-length precursor sequence, including the N-terminal signal peptide.

Congenital leptin deficiency/dysfunction is due to mutations in the *LEP* gene, which encodes the expression of leptin protein (Fig. 1 *A*) (6). Leptin is critical for regulating body weight, and the protein’s biological activity is strongly dependent on its interaction with the LepR. Once produced by adipocytes, the circulating leptin activates the LepR located on the surface of a subset of hypothalamic neurons (8,9,10). The receptor’s activation then induces the phosphorylation and subsequent activation of the transcription factor signal transducer and activator of transcription 3 (STAT3) (10). This, in turn, stimulates production of anorexigenic peptides while suppressing food intake and increasing energy expenditure (10).

Recent work by Tsirigotaki et al. (2023) showed an assembly of the human leptin-LepR signaling complex with a binding stoichiometry of 3:3 (PDB: 8AVF and 8AVO) (12) (Fig. 1, *D*–*G*). The authors proposed that the formation of this trimeric structure might involve an intermediate open 2:2 leptin-LepR complex (PDB: 8AVE) (12). In this regard, a 1:1 leptin-LepR interacts with an open 2:2 complex to form a more stable 3:3 structure. Two types of the 3:3 leptin-LepR complex structures have been reported including closed (PDB: 8AVF) (12) (Fig. 1, *D* and *E*) and open structures (PDB: 8AVO) (12) (Fig. 1, *F* and *G*). In the closed structure, each leptin molecule interacts with the cytokine-receptor homology 2 (CRH2) domain of one LepR using its α-helices A and C (binding site II) (Fig. 1 *B*). Concurrently, the molecule engages the immunoglobulin-like domain (IgD) of another LepR using the N-terminal of its α-helix D and the AB and CD loops (binding site III) (Fig. 1 *C*). Ultimately, this together forms a closed circular structure. In contrast, the open structure features one leptin molecule interacting only with the LepR CRH2 domain, whereas the other two leptin molecules interact with both the CRH2 and IgD domains.

Mutagenesis experiments indicated that the LepR CRH2 domain is a high-affinity binding site for leptin (13). Indeed, a single CRH2 domain has been shown to exhibit a similar affinity for leptin as the whole LepR extracellular region (14,15). Conversely, the interaction between leptin and the IgD is relatively weak, and the LepR lacking the IgD still retains leptin binding capability comparable to the wild-type (WT) receptor (14,16). Collectively, the LepR CRH2 domain is the most crucial binding site for leptin (16,17).

Even though the IgD confers no detectable binding affinity for leptin, the domain is still essential for the LepR activation (18). Studies have shown that removing this domain from the entire LepR extracellular region does not abolish leptin binding; however, the receptor is completely devoid of biological function (14,16). The interaction between leptin’s binding site III and the IgD drives the formation of a higher-order receptor cluster, which enables interaction between at least two membrane-proximal fibronectin III-like (FNIII) domains (12). These interactions possibly stimulate the LepR signaling by bridging the receptor’s transmembrane domains together, thus allowing the intracellular structure and associated JAK kinases to be in close proximity for cross-activation (12).

To date, a total of 28 distinct homozygous mutations in the *LEP* gene have been reported as causative for congenital leptin deficiency/dysfunction (19). Most of these mutations cause defects in leptin synthesis or secretion (6,20,21) or impaired LepR binding (22,23). For instance, the leptin mutation Leu72Ser (Fig. 1 *A*), which was first described in a child from an Austrian pedigree in 2010 (24), causes the protein to lose its stability (25). Consequently, the mutant (MT) protein is not secreted into circulation (24).

At least three mutations (Gly59Ser, Pro64Ser, and Ser141Cys) have been found not disrupting leptin synthesis, secretion, or receptor binding, but instead result in no or only marginal LepR signal transduction (19,26). Although Gly59 and Pro64 are located within leptin’s AB loop, Ser141 resides at the N-terminal of α-helix D (Fig. 1 *A*). The impact of Ser141Cys mutation on leptin-induced LepR signaling is well characterized. Leptin Ser141 was found to form strong hydrogen bonds with the hydroxyl group of LepR Tyr411 in the complex of leptin-LepR (10,12). Consequently, the substitution of serine for cysteine disrupts these hydrogen bonds, interferes with the site III-IgD interactions and eventually abolishes the receptor signaling. Although no direct contact between Gly59 and the LepR IgD has been observed, this residue is located in close proximity to the LepR IgD within the leptin-LepR assembly (Fig. 1 *C*). Therefore, it is plausible that the Gly59Ser mutation may still impact the leptin-LepR IgD binding interface and cause impaired receptor activation and the following downstream signaling.

In contrast to Gly59 and Ser141, Pro64 is located distant from the leptin-LepR IgD binding interfaces and is not involved in any interaction with the LepR residues (Fig. 1 *C*). Nevertheless, the Pro64Ser mutation exerts a more pronounced effect on leptin-induced LepR signaling than the Gly59Ser mutation (26). Although the Gly59Ser MT retains approximately 18% of the WT signaling activity, the Pro64Ser MT exhibits only ∼3% (26). To elucidate the origins of congenital leptin deficiency/dysfunction resulting from leptin mutations as well as to explore possible mechanisms of initiating signal transduction from a structural biology perspective, it is necessary to investigate the underlying reasons for the defect in intracellular signaling caused by the Pro64Ser mutation. The present study aims to address three primary objectives.

1) Identifying potential structural alterations in leptin caused by the Pro64Ser mutation.
2) Validating the effect of Pro64Ser mutation on leptin structure and its capability to bind to the LepR.
3) Establishing possible connections between any observed structural alterations and the impaired signaling capacity of MT leptin.

To achieve these objectives, we employed theoretical approaches, including multiple conventional molecular dynamics (MD) simulations of both the WT and MT Pro64Ser leptins in their bound and unbound states. We also conducted free energy computations to assess changes in leptin folding and binding after the Pro64Ser mutation. The detrimental effect of the Leu72Ser mutation on leptin’s structural integrity was well characterized (25). Hence, this mutation was used as a negative control when evaluating the impact of the Pro64Ser mutation on leptin structure in both the folding free energy computations and conventional MD simulations.

## Materials and Methods

### Research system

Two available human leptin structures in unbound state have been identified: the crystal structure of MT leptin W100E (PDB: 1AX8) (11) and the NMR structures of native leptin (PDB: 8K6Z) (27). Free leptin has been shown to rapidly degrade in acidic environments (pH 2.0) (28). The protein exhibits resistance to proteolytic conditions only when bound to the soluble LepR (28). The NMR structures of native leptin were determined under low pH conditions (pH 4.5) (27). Even though the environment was less acidic than the condition used in the research of Cammisotto and Bendayan (2012), the resolved leptin structures might still exhibit reduced stability. Thus, these structures were not chosen for the present computational study.

The crystal structure of MT leptin W100E was resolved under physiological pH (pH 7.5). Due to the extensive aggregation propensity of native protein, the W100E mutation was introduced to enhance its solubility (11). The biological activities of the WT and MT leptins were found to be comparable (11). As the MT W100E structure lacks a portion of the loop connecting α-helices A and B (∼14 amino acids), homology modeling was conducted to obtain the full-length human leptin structure. The modeling procedure is detailed below.

The human leptin amino acid sequence was retrieved from the National Center for Biotechnology Information (NCBI) (https://www.ncbi.nlm.nih.gov) using NCBI Reference Sequence of NP_000221.1. The signal peptide, which comprises the first 21 amino acids of the sequence, was removed, and the remaining sequence encoding for mature protein was submitted to the SWISS-MODEL web server (29,30,31) for homology modeling. The crystal structure of MT W100E was used as a template for the modeling procedure. For the conserved amino acids defined by target-template alignment, their atomic coordinates were generated by automatically transferring the coordinates of corresponding amino acids in the template protein structure (31). The missing portion of AB loop in the MT leptin structure were resolved using the ProMod3 modeling engine (30) integrated in the SWISS-MODEL server. The resulting model of mature leptin was used as the WT reference for the following MD simulations (Fig. 1 *A*). The amino acid numbering of WT leptin used throughout this study is based on the full-length precursor sequence, including the N-terminal signal peptide. The complete numbering scheme is provided in Fig. S1.

The LepR CRH2 domain serves as a high-affinity binding site for leptin. For computational efficiency in estimating leptin binding free energy change for the LepR upon the Pro64Ser mutation, we simplified the system by using only the monomeric leptin-LepR CRH2 (Fig. 1 *B*) derived from the 3:3 assembly of human signaling complexes (PDB: 8AVF) (12). The CRH2 domain spans amino acids 428–633 (Fig. S2).

### Free energy computations

In this study, the changes in leptin folding free energy and binding free energy for the CRH2 domain were computed following the Pro64Ser mutation. The computation of leptin folding free energy change provides insights into the mutation’s effect on the protein’s thermostability. Meanwhile, the leptin binding free energy change indicates the impact of mutation on the protein’s ability to interact with its high-affinity binding site on the LepR. The mutation Leu72Ser, which confers detrimental effect on leptin structure (25), was used as a negative control for evaluating leptin’s folding free energy change upon the Pro64Ser mutation.

Both of the free energy change computations were conducted using the PMX software package (32,33,34) combined with GROMACS 2021.2 (35). The Amber99SB^∗^ILDN force field (36) was employed in both cases to derive the interaction parameters for leptin and the LepR. The change in leptin folding free energy upon the Pro64Ser or Leu72Ser mutation was computed using the thermodynamic cycle presented in Fig. S3 *A*. The binding free energy change of leptin to the LepR CRH2 upon the Pro64Ser mutation was computed following the cycle displayed in Fig. S3 *B*.

To calculate the leptin folding free energy change ($\Delta \Delta G_{folding}^{mutation}$), the free energy taken for the WT and MT leptins to transform from their unfolded to folded states ($\Delta G_{folding}^{WT}$ and $\Delta G_{folding}^{MT}$) is required. By contrast, the free energy required for the solvated WT and MT leptins to interact with the LepR CRH2 domain ($\Delta G_{binding}^{WT}$ and $\Delta G_{binding}^{MT}$) is needed for computing the leptin binding free energy change ($\Delta \Delta G_{binding}^{mutation}$). However, the $\Delta G_{folding}^{WT}$, $\Delta G_{folding}^{MT}$, $\Delta G_{binding}^{WT}\text{,}$ and $\Delta G_{binding}^{MT}$ are inaccessible through conventional MD simulations. Meanwhile, the $\Delta G_{unfolded}^{mutation}$, $\Delta G_{folded}^{mutation}$, $\Delta G_{unbound}^{mutation}\text{,}$ and $\Delta G_{bound}^{mutation}$ are easier to compute by alchemically transforming the WT leptin amino acid to its MT state in both the forward and reverse directions. As the thermodynamic free energy is a state function, the $\Delta \Delta G_{folding}^{mutation}$ and $\Delta \Delta G_{binding}^{mutation}$ can be derived from

$$\Delta \Delta G_{folding}^{mutation}=\Delta G_{folding}^{MT}-\Delta G_{folding}^{WT}=\Delta G_{folded}^{mutation}-\Delta G_{unfolded}^{mutation}$$

and

$$\Delta \Delta G_{binding}^{mutation}=\Delta G_{binding}^{MT}-\Delta G_{binding}^{WT}=\Delta G_{bound}^{mutation}-\Delta G_{unbound}^{mutation}\text{.}$$

#### Leptin folding free energy

The leptin folding free energy change upon the Pro64Ser or Leu72Ser mutation ($\Delta \Delta G_{folding}^{mutation}$) can be estimated by evaluating the difference between the $\Delta G_{folded}^{mutation}$ and $\Delta G_{unfolded}^{mutation}$ (Fig. S3 *A*). The $\Delta G_{folded}^{mutation}$ represents the free energy difference between the folded states of WT and MT leptins. The $\Delta G_{unfolded}^{mutation}$ denotes the free energy difference between the unfolded states of WT and MT proteins.

To compute the $\Delta G_{folded}^{mutation}$, the following procedure was conducted. The hybrid structure and topology of folded leptin, which contains both the WT and MT amino acids simultaneously, were first generated using the PMX software (32,33,34). The hybrid leptin structure was placed in a dodecahedral box with at least 2.0 nm from the box edges. The system was solvated using the TIP3P water model (37) and neutralized with sodium and chloride ions at a concentration of 150 mM. In the subsequent step, two independent equilibrium simulations were performed. In one simulation, leptin has the WT amino acid (λ = 0). In another simulation, the protein has the MT amino acid (λ = 1). For each simulation, a separate energy minimization using the steepest descent algorithm was conducted. The energy-minimized configurations were then subjected to 1 ns of equilibration simulation under the isobaric-isothermal ensemble (NPT). During the equilibration simulations, position restraints were applied to all nonhydrogen atoms. Finally, the restraints were lifted, and 50 ns of equilibrium simulation was conducted for each WT and MT leptins. The Berendsen (38) and Parrinello-Rahman (39) pressure coupling was used to maintain constant pressure at 1 atom and temperature at 298.15 K during the equilibration and equilibrium simulations, respectively.

Once the equilibrium simulations were completed, nonequilibrium simulations were initiated by extracting 100 frames equidistantly from the last 30 ns of each WT and MT leptin equilibrium simulation trajectories. Each of these frames served as a starting leptin conformation for another 1 ns of nonequilibrium transition. The leptin conformations starting at λ = 0 (WT amino acid state) were alchemically transformed into λ = 1 (MT amino acid state). By contrast, the protein conformations starting at λ = 1 were transformed into λ = 0. In both the forward and reverse transformations, the λ values were transformed continuously at a speed of 2 × 10^−6^ per step. In each transformation, the derivatives of the system Hamiltonian with respect to the λ parameter were recorded and then used to estimate the associated work. Finally, we utilized the Bennett acceptance ratio estimator (40) to derive the energy difference between the WT and MT folded leptins, which corresponds to the $\Delta G_{folded}^{mutation}$.

In the context of alchemical free energy computations, the protein’s unfolded state can be approximated using a capped tripeptide in the form of GXG, where a residue of interest (X) is surrounded by two glycine (G) amino acids (40). To estimate the $\Delta G_{unfolded}^{mutation}$, the same MD setup used to determine the $\Delta G_{folded}^{mutation}$ was applied for the tripeptide’s Pro2Ser or Leu2Ser mutation. Finally, the change in the leptin folding free energy upon the Pro64Ser or Leu72Ser mutation is estimated by computing

$$\Delta \Delta G_{folding}^{mutation}=\Delta G_{folding}^{MT}-\Delta G_{folding}^{WT}=\Delta G_{folded}^{mutation}-\Delta G_{unfolded}^{mutation}\text{.}$$

To assess the reliability of the resulting $\Delta \Delta G_{folding}^{mutation}$, for each computation associated with folded or unfolded leptin, the whole procedure including energy minimization and equilibrium and nonequilibrium simulations was repeated five times.

#### Leptin binding free energy

The relative binding free energy change ($\Delta \Delta G_{binding}^{mutation}$) of leptin to the LepR CRH2 after the Pro64Ser mutation was estimated by calculating the difference between the $\Delta G_{bound}^{mutation}$ and $\Delta G_{unbound}^{mutation}$ values (Fig. S3 *B*). Here, the $\Delta G_{bound}^{mutation}$ represents the free energy difference between the WT and MT leptin when in bound states. Conversely, the $\Delta G_{unbound}^{mutation}$ is identical to the $\Delta G_{folded}^{mutation}$ described in the leptin folding free energy section above. Thus, the previously computed $\Delta G_{folded}^{mutation}$ values were used as the $\Delta G_{unbound}^{mutation}$ values for the binding free energy change computation. The computational setup for determining the $\Delta G_{bound}^{mutation}$ followed a similar procedure as explained in the leptin folding free energy section. Finally, the overall binding free energy change for leptin to the LepR CRH2 was computed as follows:

$$\Delta \Delta G_{binding}^{mutation}=\Delta G_{binding}^{MT}-\Delta G_{binding}^{WT}=\Delta G_{bound}^{mutation}-\Delta G_{unbound}^{mutation}\text{.}$$

Note that each monomeric leptin-LepR CRH2 from the 3:3 leptin-LepR assembly (PDB: 8AVF) (12) was used as the starting complex structure in two repetitions of the $\Delta G_{bound}^{mutation}$ computations. Thus, in total, six repetitions of the computations were conducted.

### Multiple ATMD simulations of WT and MT leptins

#### Leptin simulation setup

The Pro64Ser and Leu72Ser mutation effect on the leptin structure was also thoroughly investigated through multiple independent atomistic MD (ATMD) simulations of both the WT and MT proteins free in solution. Like the leptin folding free energy computations, standard simulations of the Leu72Ser leptin were used as a negative control to better estimate the impact of Pro64Ser mutation on leptin structure. First, the MT leptin structures were generated using the structure editing tool embedded in UCSF Chimera (41). The rotameric state of MT Ser64 and Ser72 were determined by choosing the one with the highest probability of occurrence within the Dunbrack rotamer library (42).

Both simulations of the WT and MT leptins were executed using GROMACS 2021.2 (35) and the Amber99SB-ILDN force field (43). The WT and MT proteins were solvated in a cubic box using the SPC/E water model (37). To maintain charge neutrality, sodium and chloride ions at a concentration of 150 mM were added to the simulated systems. The box sizes were defined such that the distance between any atoms of the simulated proteins and any box edges is at least 1.0 nm. Both systems were then energy-minimized using the steepest descent algorithm and followed by a two-phase equilibration. The first equilibration involves a 500-ps simulation under the isothermal-isochoric (NVT) ensemble. The second one entails a 1-ns simulation conducted under the isobaric-isothermal ensemble ensemble (NPT). Both equilibrations were conducted with the presence of position restraint imposed on all the protein’s nonhydrogen atoms. The production runs were performed for 100 ns, and Parrinello-Rahman pressure coupling (39) and V-rescale temperature coupling (44) were employed to maintain constant pressure at 1 atm and temperature at 300K, respectively. It is noteworthy that in each of the WT and MT leptin simulations, the whole workflow including energy minimization, two phases of equilibration, and 100-ns production run was repeated 10 times with a different random seed for the initial velocities in the NVT equilibration.

#### Leptin multiple simulations analyses

A total of 10 independent ATMD simulations (100 ns each) were performed for each of the WT and MT leptins. Each set of the simulations was then combined to generate a single atomistic trajectory that consists of 100,000 frames (10 simulations x 10,000 frames/simulation). All the analyses related to the multiple ATMD simulations of the WT and MT leptins when free in solution were conducted on their corresponding atomistic concatenated trajectories.

##### Amino acid interaction network

For each amino acid forming the mature leptin structure, an interaction center was defined. This represents the center of geometry of all nonhydrogen atoms within the amino acid’s side chain. Except for glycine, which is an amino acid containing only a hydrogen atom in its side chain, the α-carbon (Cα) atom was defined as the interaction center. Employing the Python-based library MDAnalysis (45,46), the distance between the interaction center of a selected target amino acid and the centers of all the other leptin amino acids was measured for each frame within the WT and MT concatenated trajectories. For each pair of amino acids, their initial interaction center distances were obtained by averaging the distances measured in the first frame of each individual trajectory generated from the 10 independent simulations. Finally, to visualize the distribution of sampled distances, a separate 1D probability distribution plot was generated for each pair of residues.

##### Estimation of volume enclosed by leptin helices

To assess the impact of researched mutations (Pro64Ser and Leu72Ser) on the overall leptin structure, the volume enclosed by the protein’s four major α-helices was measured for each frame of the WT and MT trajectories. The Python library SciPy (47) was utilized to define a convex hull encompassing all the Cα atoms of leptin helices followed by the calculation of its volume. Note that the convex hull of a set of coordinates in a three-dimensional space is the smallest convex polygon that encloses all of the given coordinates (48). Thus, the volume of this convex hull serves as an approximation for the volume enclosed by the protein’s helices. In the first frame of each individual trajectory (10 simulations per WT/MT), the volume of leptin helices was estimated and subsequently averaged to represent the initial volume value. Finally, separate plots were generated to visualize the protein volume distribution for the WT and MT simulations.

##### Hydrogen bond network analysis

To elucidate differences in the hydrogen bond network of a selected amino acid in the WT and MT leptin, the hydrogen bond analysis scheme designed by Ngo and Juffer (2024) was employed (49). The analysis aimed to detect all the available hydrogen bonds between the selected amino acid and its surrounding amino acids throughout the simulations. Afterward, the total number of detected hydrogen bonds formed between a pair of amino acids in the WT protein simulations was compared with that in the MT simulations to identify differences in their hydrogen bond networks.

##### Other analyses

1) The secondary structure elements of WT/MT leptin in each structural frame of their concatenated trajectories were determined using the GROMACS built-in tool gmx do_dssp. For a selected sequence of amino acids within the protein structure, the number of amino acids that are assigned as helix (α-helix, 3_10_ helix, and π-helix) was counted and divided by the total number of amino acids within the sequence to determine the helical content (expressed in a percentage). Similarly, the loop content (expressed in a percentage) was calculated by counting the number of amino acids assigned as coil and normalizing this value by the total number of amino acids in the sequence.
2) The root mean-square fluctuation (RMSF) profiles of the Cα atoms of WT and MT leptins were computed using the tool gmx rmsf.

### Multiple ATMD simulations of WT/MT leptin-LepR complexes

#### Complexes’ simulation setup

Multiple independent ATMD simulations were performed for both the WT and MT Pro64Ser leptin-LepR homotrimers. The objective was to first further investigate the impact of Pro64Ser mutation on the interactions between leptin and the LepR CRH2 within the context of the 3:3 assembly. Furthermore, we also aimed to discuss the potential effect of this mutation on the ability of MT leptin to induce higher-order clustering of the LepR, which is crucial for the receptor activation and subsequent signal transduction.

The input structure for complex simulations was derived from the cryo-EM structure of closed 3:3 human leptin-LepR (PDB: 8AVF) (12). Note that the membrane-distal cytokine receptor homology 1 (CRH1) domain is absent in the LepR extracellular region in the complex provided by Tsirigotaki et al. However, previous studies have demonstrated that the CRH1 domain is not essential for leptin binding and for the activation and signaling of the receptor (14,16). Therefore, simulating the WT/MT leptin-LepR ectodomain complex without the CRH1 domain is expected to adequately address the objectives outlined above.

In the 3:3 leptin-LepR complex provided by Tsirigotaki et al. (PDB: 8AVF) (12), three leptin molecules are designated as chains A, C, and E, respectively. Correspondingly, three LepRs are denoted as chains B, D, and F. For the rest of this article, the leptin molecules in chains A, C, and E will be referred to as Leptin1, Leptin2, and Leptin3, respectively. Similarly, the receptor molecules designated as chains B, D, and F in the homotrimeric structure will be referred to as LepR1, LepR2, and LepR3.

Given low resolution (6.45 Å) of the researched complex structure, an initial structural refinement was performed using MD simulation. This refinement was conducted employing GROMACS 2021.2 (35) and the Amber99SB-ILDN force field (43). The simulation setup, including system establishment, energy minimization, two-phase equilibrations, and a 100-ns production run, followed the procedure described for the WT/MT leptin molecules’ simulations (section leptin simulation setup). The structural refinement simulation was conducted only once.

The final frame of 100-ns refinement simulation was extracted and used as the starting structure for subsequent multiple all-atom simulations. To generate the MT complex, the Pro64Ser mutation was introduced into each leptin molecule within the refined homotrimers using the UCSF Chimera structure editing tool (41). The mutant rotameric states were selected based on the Dunbrack rotamer library (42). The ATMD simulations of both the WT and MT Pro64Ser leptin-LepR complexes were conducted following the procedure described in section leptin simulation setup and repeated 10 times for each type of complex.

#### Complexes’ simulation analyses

The trajectories generated from each type of the WT and MT leptin-LepR complexes’ simulations were concatenated to form a pseudo-trajectory comprising approximately 100,000 frames (10 simulations × 10,000 frames per simulation). All the subsequent analyses were conducted on the resulting pseudo-trajectory from each WT and MT simulations.

1) Within each monomeric leptin-LepR CRH2 or leptin-LepR IgD structure in the initial refined homotrimers, the interface amino acids between the two molecules were identified using the PyMOL “InterfaceResidues” script (https://pymolwiki.org/index.php/InterfaceResidues). Afterward, the distance between the center of geometry of leptin interface residues and the LepR CRH2/IgD interface residues (Fig. S4) was measured for each frame within the concatenated trajectories using the Python-based library MDAnalysis (45,46).
2) The root mean-square deviation (RMSD) values for all the Cα atoms from the amino acids that belong to the WT/MT leptin molecule and the LepR CRH2 or IgD (relative to the refined leptin-LepR structure) were computed using the GROMACS tool gmx rms.

## Results

The results are presented as follows. Section structural impact of mutations on leptin investigates the structural consequences of Pro64Ser mutation in leptin using folding free energy change computations and multiple atomistic MD simulations. The mutation Leu72Ser, which is known to exert a structurally destabilizing effect, will be used as a negative control for this investigation. Section functional impact of Pro64ser mutation on leptin explores the impact of Pro64Ser substitution on leptin’s binding affinity toward its high-affinity binding site on the LepR CRH2 domain using binding free energy calculations. Specifically, this section elucidates potential molecular mechanisms underlying the inability of the MT leptin to activate LepR-mediated intracellular signaling.

### Structural impact of mutations on leptin

#### Leptin folding free energy change upon mutation

To evaluate the impacts of Pro64Ser and Leu72Ser mutations on leptin stability, five independent computations of protein folding free energy change were conducted for each mutation. The results of these computations are summarized in Table 1. The average free energy change after the Leu72Ser mutation was determined to be $\Delta \Delta G_{folding}^{Leu72Ser}$ = 32.48 kJ/mol (7.76 kcal/mol) with a standard error of approximately 0.40 kJ/mol. This finding is consistent with the experimental data demonstrating that the Leu72Ser mutation severely lowers leptin stability (25). This destabilizing effect likely prevents the MT leptin from maintaining its native conformation, which explains why the protein fails to reach circulation despite successful synthesis (24).

**Table 1:** Free energy difference (ΔG) associated with alchemically transforming the WT into MT amino acid in both forward and reverse directions, following the procedure described in section free energy computations

	$\Delta G_{folded}^{Leu72Ser}$ (kJ/mol)	$\Delta G_{unfolded}^{Leu72Ser}$ (kJ/mol)	$\Delta G_{folded}^{Pro64Ser}$ (kJ/mol)	$\Delta G_{unfolded}^{Pro64Ser}$ (kJ/mol)	$\Delta G_{bound}^{Pro64Ser}$ (kJ/mol)	$\Delta G_{unbound}^{Pro64Ser}$ (kJ/mol)
Run1	69.12 ± 0.52	34.36 ± 0.15	−173.04 ± 0.45	−173.98 ± 0.50	−159.17 ± 0.63	−173.04 ± 0.45
Run2	69.27 ± 0.51	34.10 ± 0.17	−174.68 ± 0.35	−172.73 ± 0.43	−161.77 ± 0.43	−174.68 ± 0.35
Run3	62.54 ± 0.42	34.51 ± 0.15	−182.96 ± 0.53	−173.96 ± 0.38	−164.14 ± 0.49	−182.96 ± 0.53
Run4	63.34 ± 0.62	33.86 ± 0.15	−176.72 ± 0.42	−173.80 ± 0.42	−165.50 ± 0.62	−176.72 ± 0.42
Run5	69.50 ± 0.56	34.51 ± 0.21	−176.35 ± 0.42	−173.21 ± 0.44	−162.54 ± 0.60	−176.35 ± 0.42
Run6	–	–	–	–	−158.73 ± 0.40	–
Average	66.75 ± 0.53	34.27 ± 0.17	−176.75 ± 0.44	−173.54 ± 0.19	−161.98 ± 0.54	−176.75 ± 0.44

The $\Delta G_{folded}^{Pro64Ser}$ and $\Delta G_{folded}^{Leu72Ser}$ represent the free energy differences between the WT and MT leptins (Leu72Ser or Pro64Ser) in folded states. The $\Delta G_{unfolded}^{Pro64Ser}$ and $\Delta G_{unfolded}^{Leu72Ser}$ denote the free energy differences between the WT and MT unfolded leptins. The capped tripeptides (GXG) were used to approximate the unfolded proteins. The $\Delta G_{bound}^{Pro64Ser}$ and $\Delta G_{unbound}^{Pro64Ser}$ correspond to the free energy differences between the WT and MT leptins when in bound and unbound states, respectively. Note that the $\Delta G_{unbound}^{Pro64Ser}$ is equivalent to the $\Delta G_{folded}^{Pro64Ser}$, as both refer to the folded leptin free in solution.

In contrast, the Pro64Ser mutation exhibits a stabilizing effect on leptin stability. The computed $\Delta \Delta G_{folding}^{Pro64Ser}$ of −3.21 kJ/mol (−0.77 kcal/mol) (standard error: ∼0.34 kJ/mol) indicates an enhancement of the protein stability after the introduction of Pro64Ser mutation. This observation also aligns very well with the previous report of comparable circulating levels of the WT and MT leptins (26).

#### Detailed investigation of the MT leptin structure

**Figure 2:**
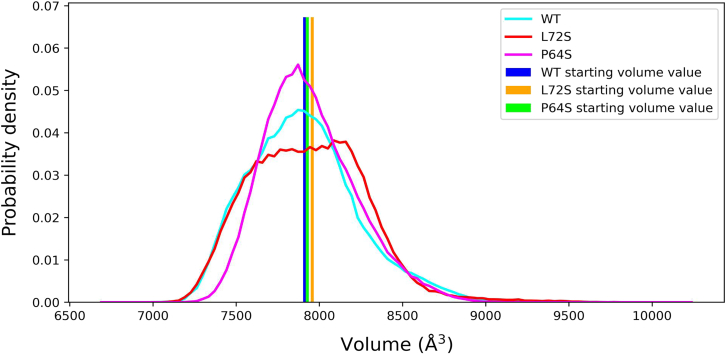
The probability distributions of helical core volumes for the WT leptin and the Pro64Ser and Leu72Ser MTs. The distribution curves for the WT, Pro64Ser, and Leu72Ser leptins are depicted in cyan, magenta, and red, respectively. Initial volume values for the WT, Pro64Ser, and Leu72Ser are shown as bars and colored blue, orange, and green, respectively.

To study the effect of each of the Pro64Ser and Leu72Ser mutations on the leptin structure in greater detail, we performed multiple independent ATMD simulations for the WT and MT leptins in their unbound states. Leptin’s tertiary structure is characterized by four major antiparallel α-helices. Hence, the volume enclosed by these helices was measured across the WT and MT concatenated trajectories to assess the structural impact of each mutation. The results of these measurements were presented in Fig. 2. Here, the helical core volume within the WT and MT Pro64Ser leptin structures exhibits a distinct peak near the initial volume value (less than 8000 Å^3^). By contrast, the sampled volume values for region within the Leu72Ser leptin’s helices display a uniform distribution (ranging from ∼7500 to 8200 Å^3^).

The observations suggest a frequent expansion and contraction of the environment inside the four α-helices within the Leu72Ser MT structure, potentially disrupting the intrahelical interactions. This disruption may represent the initial stage of this MT protein’s denaturation process, which eventually leads to protein misfolding and impaired secretion into circulation (25). Conversely, the multihelical conformations within the WT and Pro64Ser leptin structures remained stable throughout the simulations, as indicated by their bell-shaped volume distributions.

**Figure 3:**
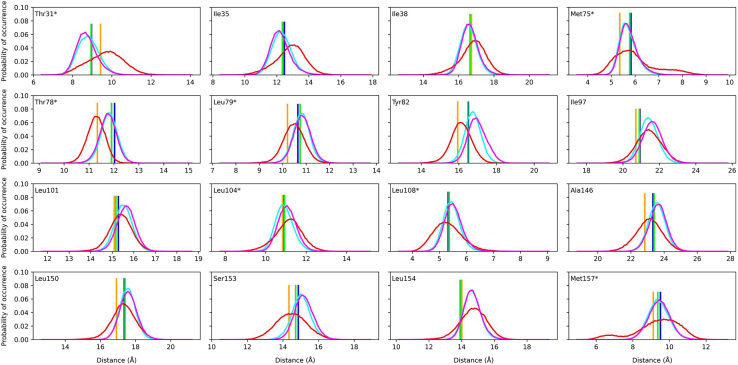
Probability distributions of the side chain center-of-geometry distances between amino acid 72 and the other fully buried amino acids in the WT and MT Pro64Ser and Leu72Ser leptin structures. The buried amino acids were determined based on their SASA values. The amino acids that are in close proximity to the WT Leu72 are denoted by an asterisk (^∗^) adjacent to their residue IDs. The distribution curves for the distance involving residue 72 within the WT, Pro64Ser, and Leu72Ser leptin structure are colored cyan, magenta, and red, respectively. The WT and MT initial distances (averaged across the first structural frames in each of the 10 independent WT/MT trajectories) are represented as bars and colored blue, green, and orange for the WT, Pro64Ser, and Leu72Ser leptin structures, respectively.

**Figure 4:**
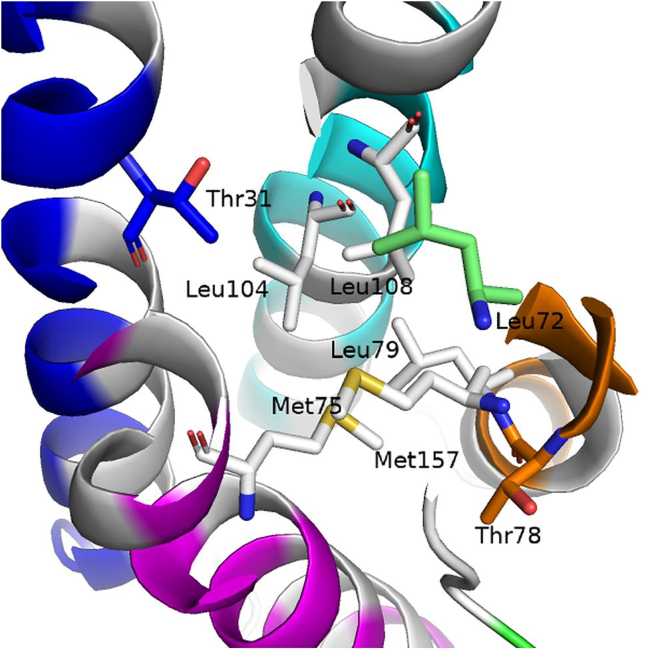
The amino acids that are fully buried in the leptin core and located in close proximity to the WT Leu72 (*shown as lime green stick*). These amino acids are depicted as sticks. Specifically, the amino acids with hydrophobic side chains are colored white. The polar amino acids located on the helices A, B, C, and D are colored blue, orange, cyan, and magenta, respectively. The figure was generated using PyMOL Molecular Graphics System (open source, Schrödinger, LLC).

To further clarify underlying reasons for the enhanced flexibility of helical core in the Leu72Ser leptin compared with the WT and Pro64Ser variants, the interaction network involving amino acid 72 was inspected throughout the WT and MT atomistic trajectories. Fig. 3 depicts the probability distributions of distances between the interaction center of amino acid 72 and those of the amino acids that are fully buried in the leptin core. Table S1 summarizes the statistical measures derived from each distribution curve shown in Fig. 3. To determine the fully buried residues within leptin core, the solvent accessible surface area (SASA) was calculated for each of the WT leptin amino acids using PyMOL Molecular Graphics System (open source, Schrödinger). The analysis revealed a group of 17 amino acids including Thr31, Ile35, Ile38, Leu72, Met75, Thr78, Leu79, Tyr82, Ile97, Leu101, Leu104, Leu108, Ala146, Leu150, Ser153, Leu154, and Met157 being entirely buried (SASA value of 0.0). Among those amino acids, seven (Thr31, Met75, Thr78, Leu79, Leu104, Leu108, and Met157) are located in close proximity to the WT Leu72 (Fig. 4). These amino acids possess at least one atom that is within 8 Å of any of the atoms from the WT Leu72.

In general, the distribution curves for distances between amino acid 72 and the fully buried amino acids in the Leu72Ser leptin structure show larger variance compared with the corresponding distances in the WT and Pro64Ser leptins (Fig. 3; Table S1). In addition, for each inspected pair of amino acids, the WT/Pro64Ser simulations also exhibit a greater proportion of structural frames, where the distance between the residue 72 and the examined residue remains within 5% of their initial value (Fig. 3; Table S1). The presented data illustrate a stable distance between amino acid 72’s side chain and the side chain of any examined amino acid throughout the WT and Pro64Ser leptins simulations. By contrast, the measured distances present greater variability during the Leu72Ser leptin simulations.

Note that 14 out of the 17 fully buried amino acids in leptin’s core possess hydrophobic side chains (Ile35, Ile38, Leu72, Met75, Leu79, Tyr82, Ile97, Leu101, Leu104, Leu108, Ala146, Leu150, Leu154, and Met157). Taken together, our findings strongly suggest that substituting a hydrophobic amino acid (Leu) for a polar amino acid (Ser) disrupts the network of hydrophobic interactions within the core of leptin. This disruption likely accounts for the unstable environment surrounded by the four α-helices within the MT Leu72Ser leptin structure and thus reduced protein stability. Conversely, the side chain of WT Pro64 is oriented toward the solvent environment (Fig. 1 *A*). Therefore, a hydrophobic-to-polar amino acid substitution (Pro64Ser) does not cause any disruption to the hydrophobic environment within the helices. To conclude, our simulation results indicate a strong destabilizing effect of the Leu72Ser mutation, whereas the Pro64Ser mutation does not confer any significant impact on the structural integrity of leptin. These findings are consistent with the presented folding free energy computations and available experimental data (25,26).

### Functional impact of Pro64Ser mutation on leptin

#### MT leptin binding to the CRH2 domain

The LepR CRH2 domain is primarily responsible for the binding affinity to leptin (14,15). Thus, the binding free energies of WT and MT Pro64Ser leptins to the CRH2 domain were compared to assess the effect of Pro64Ser mutation on the capability of leptin to interact with the LepR. Table 1 shows that the average change in binding free energy upon the mutation equals to $\Delta \Delta G_{binding}^{Pro64Ser}$ = 14.77 kJ/mol or 3.53 kcal/mol (standard error of ∼0.44 kJ/mol). The result underscores a significant decreased binding affinity of the MT leptin to the CRH2 domain. This finding contradicts the experimental data, which demonstrated similar equilibrium dissociation constants (K_d_) for the WT and Pro64Ser leptins when binding to the isolated monomeric CRH2 domain (26).

The computation of leptin binding free energy change upon the Pro64Ser mutation requires the simulations of WT and MT leptins in bound and unbound states (section free energy computations). Each system of the WT and MT leptin-LepR CRH2 underwent 50 ns of equilibrium MD simulation, followed by 1 ns of nonequilibrium alchemical transitions for each extracted structural frame. These simulation parameters were selected based on data presented above, which shows that similar timescales are sufficient to capture the effects of Pro64Ser and Leu72Ser mutations on leptin stability. To rule out insufficient local sampling as the source of contradiction between the experimental and computational results, we extended the equilibrium simulations (from 50 ns to 500 ns) and the nonequilibrium transition of each extracted structural frame (from 1 to 5 ns) for both the simulations of bound and unbound leptins in Run1 (Table 1). The rationale for extending equilibrium simulation times lies in the need to adequately sample the complex’s relevant conformational changes induced by the Pro64Ser mutation in the equilibrium simulations. Longer simulations increase the likelihood of capturing rare conformational transitions, thereby enhancing the accuracy of free energy calculations (32). Additionally, extending the nonequilibrium transformation period minimizes deviations from equilibrium states and reduces dissipated work along the alchemical path, further improving computational precision (32). The resulting $\Delta G_{unbound}^{Leu64Ser}=$ −178.05 ± 0.60 kJ/mol and $\Delta G_{bound}^{Leu64Ser}=$ −164.44 ± 0.63 kJ/mol remain within the range of our initial free energy estimates (Table 1). This suggests that insufficient local sampling is unlikely to be the primary cause of discrepancy between the computational and experimental observations.

#### Capability of the MT leptin to induce LepR signal transduction

**Figure 5:**
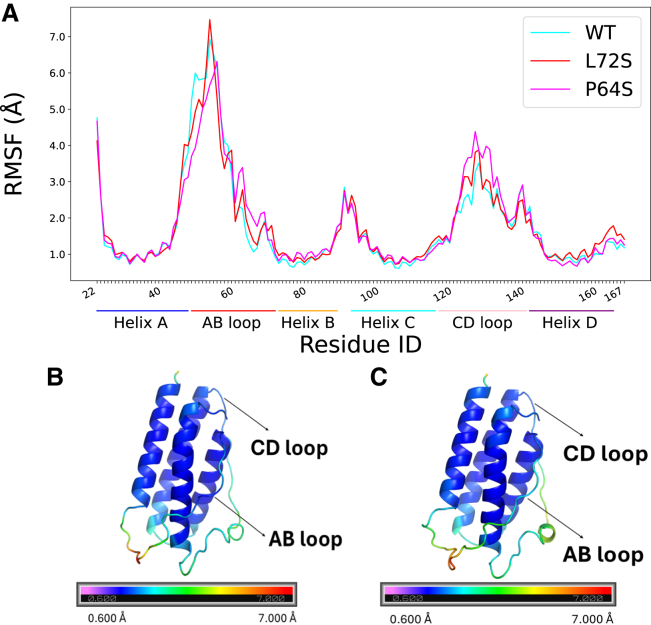
Residue-level flexibility of WT and MT leptins. (*A*) Root mean-square fluctuation (RMSF) profile of the WT and MT Pro64Ser and Leu72Ser leptins, generated from the unbound leptins’ simulations. The WT and MT Pro64Ser and Leu72Ser proteins’ RMSF curves are colored cyan, magenta, and red, respectively. A region exhibiting high RMSF values indicates significant deviations from the average positions and reflects high structural mobility. Conversely, regions with low RMSF values demonstrate minimal deviation from the average positions, thereby suggesting greater rigidity during the simulation. (*B and C*) Structural mapping of residue flexibility onto the average leptin conformations from the WT (*B*) and MT Pro64Ser (*C*) simulations. Residues are color-coded by RMSF magnitude: blue indicates low flexibility (low RMSF, in Å), and red denotes high flexibility (high RMSF). The average leptin structures were generated using the GROMACS tool gmx covar. The subfigures (B and C) were generated using PyMOL Molecular Graphics System (open source, Schrödinger, LLC). The PyMOL sessions for residue flexibility mapping on the WT and MT Pro64Ser leptins are available at https://github.com/quocbaongo/Leptin_LepR_Research/tree/b256e29279705ac965c1015c1e1655b653eb80f4/MultiSimulations_Result_and_Analysis/Multi_WT_MT_leptin_Simulations/Python_Plotting_Scripts/RMSFCA.

Previous study demonstrated that the Pro64Ser mutation, though not affecting leptin’s binding affinity to the LepR, abolishes the protein’s ability to activate the receptor and initiate intracellular signaling cascades (26). To investigate the molecular basis of this phenomenon, we conducted multiple independent MD simulations for both the WT and MT Pro64Ser leptins in their bound and unbound states. In addition, the ATMD simulations of MT Leu72Ser leptin free in solution were also performed for control purpose. Fig. 5 presents the fluctuation profiles of each amino acid within the WT and MT leptin structures, which were generated from the simulations of unbound proteins. Analysis of these profiles revealed that the N-terminal region of AB loop (approximately from Ile45 to Thr58) exhibits reduced flexibility in the MT Pro64Ser structure compared with the WT. Conversely, the C-terminal region of AB loop (approximately from Asp61 to Ile69) and the CD loop (Ser116 to Tyr140) display increased flexibility in the Pro64Ser leptin structure. Notably, the RMSF profiles of WT and Leu72Ser leptins are relatively similar, indicating that the observed alterations in loop dynamics in the MT Pro64Ser are primarily attributed to the presence of MT Ser64 residue.

The presented findings are particularly significant given that both the AB and CD loops are involved in interacting with the LepR IgD to form a higher-order leptin-LepR assembly that is competent for signaling (12). The following sections will provide detailed comparisons of these loops' conformations in the WT and MT Pro64Ser leptins. The analysis aims to provide a mechanistic link between the observed alterations in loop flexibility and impaired capacity of the MT leptin to induce LepR intracellular signaling.

##### Increased rigidity of the MT AB loop N-terminus

**Figure 6:**
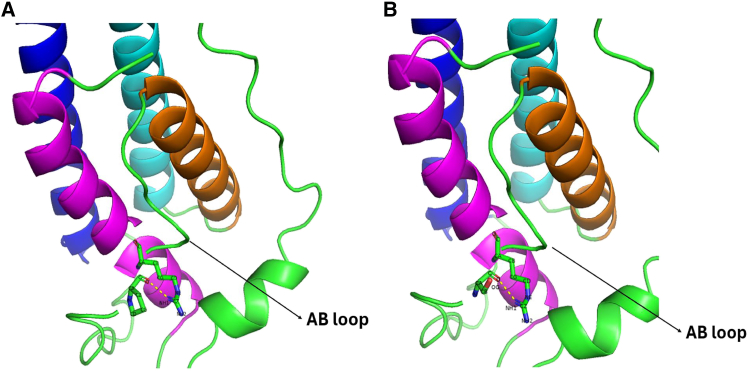
Illustration of the predominant hydrogen bond contacts between Pro64 and Arg149 and between Ser64 and Arg149, during the free WT and Pro64Ser leptin simulations, respectively. The WT Pro64, MT Ser64, and Arg149 are depicted as sticks and colored green. The atoms involved in hydrogen bonding between the two amino acids are connected by yellow dashed lines. The α-helices A, B, C, and D are shown in in blue, orange, cyan, and magenta The figure was generated using PyMOL Molecular Graphics System (open source, Schrödinger, LLC).

Hydrogen bond network analysis, following the methodology outlined by Ngo and Juffer (2024) (49), was performed on residue 64 throughout the simulations of both the free WT and MT Pro64Ser leptins to identify differences in their hydrogen bonding patterns. From Tables S2 and S3, it is evident that within the leptin structures, both WT Pro64 and MT Ser64 predominantly interact with Arg149, which is in α-helix D, via a side chain-backbone (SC-BB) hydrogen bond (H-bond) (NH1 Arg149······ O Pro64/Ser64) (Fig. 6). However, although the Arg149-Pro64 H-bond contact is observed in above 60% of frames in the WT concatenated trajectory, the Arg149-Ser64 contact is present in less than 50% of frames in the MT trajectory. This suggests a reduced frequency of this specific H-bond contact within the MT Pro64Ser leptin structure. Note that proline is an amino acid with hydrophobic side chain, and serine is a polar amino acid. The proline-to-serine substitution in the MT structure might facilitate the formation of additional H-bonds between the MT Ser64 and the surrounding solvent environment compared with the WT Pro64. Consequently, this substitution contributed to a partial disruption of the SC-BB H-bond between the MT Ser64 and Arg149 and caused a shift in the AB loop’s conformation. The consequence of this shift was an increased flexibility of the C-terminal part of AB loop. Particularly, the shift resulted in an increased rigidity of the loop N-terminus, which directly interacts with the LepR IgD in the higher-order leptin-LepR assembly.

Hydrogen bond analysis was further extended to each amino acid at the N-terminus of AB loop (residues Ile45 to Thr58), a region exhibiting increased rigidity in the MT leptin. The objective was to establish a connection between the impact of chemical property change at residue 64 and the alteration in flexibility of the MT AB loop N-terminus compared with the WT. The total number of H-bonds formed between each of these AB loop residues and any amino acids within the α-helices was counted and is summarized in Table 2. In general, it is more frequent to observe the inspected H-bond interactions in the MT structure. This suggests more frequent hydrogen bonding between the loop’s N-terminal region and the α-helices and explains why this loop portion is more rigid in the MT leptin (Fig. 5 *A*).

**Table 2:** Total number of H-bonds between each amino acid from the N-terminus of leptin’s AB loop and any amino acids within leptin’s α-helices, which were detected in the concatenated trajectories from the WT and MT Pro64Ser leptin simulations

Residue ID	Number of H-bonds with α-helices in WT Leptin Structure (out of 100,000 Total Frames)	Number of H-bonds with α-helices in MT Pro64Ser Leptin Structure (out of 100,000 Total Frames)
Ile45	1357	47
Ser46	60,298	69,374
His47	19,736	21,304
Thr48	11,060	22,954
Gln49	7192	21,904
Ser50	16,070	17,825
Val51	913	3633
Ser52	10,991	11,440
Ser53	6504	8564
Lys54	6001	3377
Gln55	4055	8554
Lys56	2230	8522
Val57	2524	174
Thr58	6617	1508

Leptin’s AB loop has been shown to be highly flexible experimentally (26,50). Indeed, our simulation data indicate that this loop is the most flexible region within the leptin structure (Fig. 5 *A*). To be signaling competent, the loop needs to undergo a conformational change to interact with the LepR IgD domain and to participate in forming the higher-order leptin-LepR assembly (10,26,51,52,53). The increased rigidity of MT leptin’s AB loop N-terminus due to enhanced hydrogen bonding with the α-helical structures might prevent this conformational change. Therefore, this likely hinders the optimal interactions between the MT’s binding site III and the IgD domain. This hypothesis provides a possible explanation for the inability of MT Pro64Ser leptin to stimulate the LepR signal transduction, despite retaining binding affinity to the CRH2 domain.

##### Increased flexibility of the MT’s CD loop

**Figure 7:**
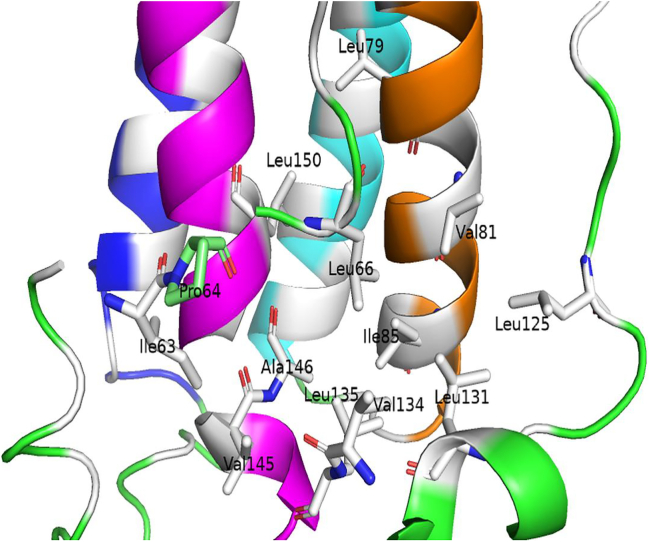
Depiction of Leu66 and a group of hydrophobic amino acids, each containing at least one atom that is within 8 Å of any atoms of Leu66 in the WT leptin structure. These amino acids are shown as sticks and colored white. Other hydrophobic residues that are not in close proximity to Leu66 are also colored white but not shown in stick representation. The WT Pro64 (mutation site) is depicted as a stick and colored lime green. The polar amino acids that belong to the helices A, B, C, and D are colored blue, orange, cyan, and magenta, respectively. The figure was generated using PyMOL Molecular Graphics System (open source, Schrödinger, LLC).

In contrast to the AB loop N-terminus, the CD loop exhibits increased mobility throughout the free Pro64Ser leptin simulations compared with the WT simulations (Fig. 5 *A*). Examining the mutation site (Pro64) within the WT leptin structure revealed a group of hydrophobic residues surrounding this amino acid including Ile63, Leu66, Leu79, Val81, Ile85, Leu125, Leu131, Val134, Leu135, Val145, Ala146, and Leu150. Notably, the side chain of Leu66 inserts into a cluster of hydrophobic amino acids located in the leptin’s CD loop and the adjacent α-helices B and D including Leu125, Leu131, Val134, Leu135, Val145, Ala146, and Leu150 (Fig. 7). This observation hints that the proline-to-serine substitution at residue 64 might disrupt the hydrophobic interactions between Leu66 and the hydrophobic residues within the CD loop and helices B and D.

**Figure 8:**
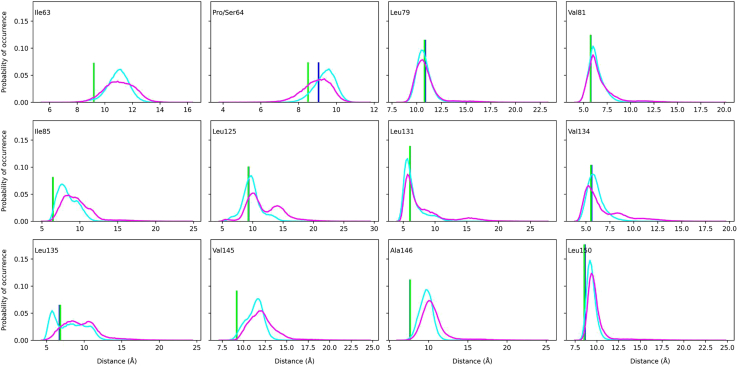
Probability distributions of the side chain center-of-geometry distances between Leu66 and surrounding hydrophobic amino acids (Ile63, Pro/Ser64, Leu79, Val81, Ile85, Leu125, Leu131, Val134, Leu135, Val145, Ala146, and Leu150) in the WT and Pro64Ser MT leptins. The distribution curves for the distances involving Leu66 in the WT and MT structures are shown in cyan and magenta, respectively. The initial distances for the WT and MT (averaged across the first structural frames of 10 independent WT/MT trajectories) are represented as bars, colored blue for the WT and green for the MT leptin structures. Note that for certain residue pairs involving Ile63, Ile85, Leu131, and Val145, the WT bars are not visible due to complete overlap with the corresponding MT bars.

To verify the aforementioned hypothesis, an amino acid interaction network analysis was performed using Leu66 as target amino acid. Specifically, the center-of-geometry distances between the side chains of Leu66 and each of the presented hydrophobic residues (Ile63, Leu66, Leu79, Val81, Ile85, Leu125, Leu131, Val134, Leu135, Val145, Ala146, and Leu150) were monitored throughout the WT and MT concatenated trajectories. As shown in Fig. 8 and Table S4, the distance distribution between Pro64 and Leu66 side chains in the WT exhibits significantly smaller variance compared with the Ser64-Leu66 distance distribution in the MT. Additionally, the number of structural frames, in which the side chain distance between these two amino acids remains within 5% of their initial distance, is greater in the WT trajectory. These findings indicate a stable hydrophobic contact between Pro64-Leu66 within the WT protein. The proline-to-serine substitution at residue 64 alters the chemical property of this amino acid and partly disrupts its hydrophobic contact with Leu66 in the MT structure.

The disruption in hydrophobic contact is not limited to only the interaction between Pro64 and Leu66 but also extends to the interactions between Leu66 and the other hydrophobic amino acids located in the CD loop and α-helices B and D (Fig. 7). The distance distributions involving the MT Ser64 show larger variance and smaller number of frames, where the measured side chains distance is within 5% of their initial distance. The disruption of Pro64-Leu66 contact upon the Pro64Ser mutation possibly leads to a disturbance in the entire hydrophobic network involving the Leu66, α-helix D and CD loop in the MT leptin structure. This hypothesis explains the increased flexibility of both the CD loop and the C-terminus of AB loop in the MT leptin (Fig. 5 *A*).

When comparing the leptin conformations in unbound (modeled leptin using the MT leptin structure, PDB: 1AX8) and bound state (PDB: 8AVF, 8AVO, 8DH9, 8X80, and 8X81), it is apparent that a portion of the protein’s CD loop (from Asp129 to Tyr140) remains as an α-helix in the bound state. Therefore, it is likely that this loop segment must undergo a transition to a more rigid helical conformation to facilitate the interactions between leptin and the LepR IgD. Consequently, the enhanced flexibility of the CD loop in the MT leptin (Fig. 5 *A*) might hinder this structural transition and impede optimal interactions between leptin molecule and the IgD from the LepR.

##### Helical and loop content of leptin’s CD loop

To validate our hypothesis regarding the ability of leptin’s CD loop C-terminus to transition to a helical state, we computed the helical content of this region for each leptin molecule when interacting with the LepR (PDB: 8AVF, 8AVO, 8DH9, 8X8O, and 8X81) (Table S5). For the sequence of 12 amino acids (from Asp129 to Tyr140), the content ranges from 69.2% to 100% (Table S5), where leptin3 in the structure of the leptin-LepR trimer (PDB: 8X80) represents the minimum helical content (∼69.2%) required for the C-terminus of CD loop to engage in interaction with the IgD (Table S5).

The helical content of each CD loop C-terminus (from Asp129 to Tyr140) was also tracked throughout the free WT and Pro64Ser leptin simulations (Fig. S5). Analyzing the WT and MT concatenated trajectories revealed a limited number of frames in both trajectories (0.160% for the WT and 0.153% for the MT), in which the helical content of CD loop C-terminus is equal to or greater than 60%. Note that the helical content of corresponding CD loop portion in the starting structure is approximately 53.84%. Thus, the result confirms the inherent capability of this loop to transform into helical structure.

Within the scope of simulations conducted in this study, we did not notice any significant difference in the ability of CD loop C-terminus within the WT and MT leptins to refold into helices. However, we found that the preexisting helices within the MT loop exhibit a stronger tendency to unfold compared with those in the WT. Specifically, 43.60% of frames within the MT concatenated trajectory display a loop content greater than 30%, whereas the corresponding figure in the WT trajectory is only 36.63%. Note that the loop content for leptin structure model used in the free leptin MD simulations is approximately 23%. This result further corroborates the increased flexibility of CD loop within the MT structure (Fig. 5 *A*).

##### Effect of Pro64Ser within the 3:3 leptin-LepR

Besides simulating the free WT and Pro64Ser leptins, both were also simulated when being within the 3:3 leptin-LepR assembly. The center-of-geometry binding interfacial distances and RMSD values of the leptin-CRH2 monomeric complexes within the higher-order leptin-LepR were collected and are presented in Fig. S6 *A* and *B*. Here, insignificant differences can be observed in the shape of the interfacial distance and RMSD distribution curves for both the WT and MT complexes. This observation is further supported by the similar mean distance values (7.69 Å for WT and 7.65 Å for MT; difference of ∼0.04 Å) and variances (0.12 Å^2^ for WT and 0.09 Å^2^ for MT) of the two distributions (Table S6). Nevertheless, the simulation outcomes have not yet allowed us to discern the Pro64Ser mutation’s impact on leptin’s binding capability to its major binding site on the LepR CRH2 domain. As the two simulations were initiated from the fully assembled 3:3 WT/MT leptin-LepR complexes, both the WT and MT leptins were already prepositioned in the conformations optimal for interacting with both the LepR CRH2 and IgD. Such prepositioning likely constrained leptin from undergoing any significant conformational changes that might have arisen due to the Pro64Ser mutation.

Similar to the leptin-CRH2 interactions, the binding interfacial distance and RMSD values distribution for the leptin-IgD’s interactions show a clear correspondence between the WT and MT complexes (Fig. S6, *C* and *D*). However, the distributions associated with the WT protein exhibit significantly larger variance, which suggest a specific degree of flexibility in the contacts between the WT leptin and LepR IgD (Table S6). This observation is consistent with the previous reports of weak binding affinity between these molecules (14,16). Conversely, the smaller variances in distributions involving the MT leptin indicate a more rigid complex structure. This rigidity is likely attributed to the C-terminal part of the rigid AB loop within the MT structure, as discussed earlier.

## Discussion

Congenital leptin deficiency or dysfunction is a rare inherited disorder that disrupts the regulation of energy homeostasis, appetite, and fat storage. One major cause of this condition is the presence of mutations that impair the proper folding of leptin protein, leading to its structural instability. A representative example is the Leu72Ser mutation. Our MD simulation data suggest that this mutation destabilizes the internal environment of the four major α-helices in the MT leptin. However, no unfolding events were observed within the simulation timescales (Fig. S5 *C*). This highlights a well-known limitation of ATMD simulations in capturing large-scale conformational transitions within accessible timescales. To overcome this, enhanced sampling techniques such as replica exchange or metadynamics (54,55,56,57) may be required to simulate unfolding processes more effectively.

Another class of pathogenic mutations affects leptin’s ability to activate the LepR-mediated intracellular signaling, despite retaining the receptor-binding capability. Among these, Gly59Ser, Pro64Ser, and Ser114Cys have been reported to impair or abolish the signaling activity. Notably, Gly59 and Ser114 are located near the interface between leptin and the IgD of LepR (Fig. S7), suggesting a direct role in receptor engagement. The Ser141 residue of leptin has been proposed to form stabilizing hydrogen bonds with the hydroxyl group of LepR Tyr411 and the carbonyl group of LepR His420 (10,12). A mutation at this site, such as Ser141Cys, could disrupt these critical interactions, thereby impairing the receptor binding and downstream signaling. This hypothesis is supported by previous studies showing that the Ser141Ala variant also fails to activate LepR signaling (58).

Gly59 itself may not directly contact LepR due to its minimal side chain; however, substitution with Serine introduces a polar side chain capable of forming new interactions. Here, Ser59 may form a polar contact with the LepR His420, potentially disrupting the native hydrogen bond network involving Ser141 (Fig. S7). This could explain the reduced signaling capacity of the Gly59Ser MT. To validate the proposed hypotheses, future simulations of the full leptin-LepR complex with a 3:3 stoichiometry, combined with detailed interaction analyses such as hydrogen bond network analysis (49), would be valuable.

In contrast to Gly59Ser and Ser141Cys, the Pro64Ser mutation is located distal to the leptin-LepR IgD interface. This mutation appears to impair signaling through an indirect mechanism. The simulation data present two potential mechanisms for this impairment. Firstly, the change in chemical property from hydrophobic to polar at residue 64 might enhance the MT amino acid’s interactions with the surrounding aqueous environment, thus altering the AB loop’s dynamics and increasing its N-terminal rigidity. This rigidity could hinder the loop’s structural rearrangements required for interacting with the LepR IgD.

Secondly, the Pro64Ser mutation increases the flexibility of CD loop by disrupting the hydrophobic contacts between Leu66 and a group of close by hydrophobic amino acids. The observed increased flexibility could prevent the C-terminus of CD loop from adopting a helical structure, which is possibly critical for leptin to engage in interactions with the IgD. Collectively, the altered dynamics of both the AB and CD loops in the MT leptin possibly impair the protein’s ability to form optimal interactions with the IgD. Hence, this disrupts the MT protein’s capacity to drive the formation of a higher-order leptin-LepR cluster, which is critical for subsequent intracellular signaling. To validate the proposed mechanisms, experimental techniques such as hydrogen/deuterium exchange mass spectrometry (59) could be employed to assess changes in protein flexibility upon mutation. Hydrogen/deuterium exchange mass spectrometry has proven to be effective in detecting mutation-induced alterations in protein dynamics (60,61,62).

Although our simulations of WT and Pro64Ser leptins provide a plausible explanation for the loss of signaling function, the computed binding free energy changes for the Pro64Ser MT contradict the experimental data. Funcke et al. (2023) reported similar binding affinities for the WT and Pro64Ser leptins to the isolated monomeric CRH2 domain. This discrepancy may stem from differences in experimental conditions and computational assumptions. The experimental setup involved immobilized monomeric CRH2 in the absence of other LepR domains, potentially allowing leptin to adopt alternative binding conformations that are not constrained by the full receptor architecture. In contrast, our free energy calculations assumed that the leptin-CRH2 conformation in the monomeric state is similar to that in the higher-order leptin-LepR complex. Notably, the 500-ns equilibrium simulation did not capture any major conformational rearrangements in the binding interface (Fig. S8). This suggests that the simulation still got stuck in a local minimum, and thus, no alternative binding modes were discovered. To conclude, our preassumption may have contributed to the observed discrepancy between experimental and computational data.

To explore potential binding conformations of leptin to the isolated CRH2 domain of the LepR, we performed protein-protein docking using the HADDOCK2.4 web server (63,64). Among the top three predicted complexes, which were ranked by HADDOCK score, leptin consistently adopts a binding orientation that aligns its interface in parallel with the CRH2 domain surface (Fig. S9, *A–C*). Notably, the leptin conformation that is the most structurally similar to that observed in the 3:3 leptin-LepR assembly (PDB: 8AVF) (12) was only ranked seventh among the 10 predicted binding modes (Fig. S9 *D*). This observation suggests that, in the absence of spatial constraints imposed by the neighboring LepR subunits, leptin tends to adopt alternative binding orientations when interacting with the isolated CRH2 domain. To further validate this hypothesis, enhanced sampling methods such as replica exchange MD (54,55,56) could be employed to simulate the monomeric leptin-CRH2 complex with initial binding conformations derived from either docking predictions or the 3:3 assembly. By allowing the system to easily overcome energy barriers and access rare conformational states, such techniques may reveal alternative binding modes and reconcile computational predictions with experimental observations.

## Data and code availability

Materials for setting up the MD simulations and conducting follow-up analyses are available at https://github.com/quocbaongo/Leptin_LepR_Research.

## Acknowledgments

We thank the center of scientific computing (CSC, https://csc.fi/) for providing us the computational resources to complete the present study, and we acknowledge 10.13039/501100013840Biocenter Finland for support.

## Author contributions

B.Q.N.: conducted computational work and follow-up data analysis, generated the figures, and wrote the manuscript. O.L.: advised research strategies and data analysis and gave feedback for the manuscript. A.H.J.: conceptualized the research project, provided funds for the project, and reviewed and gave feedback for the manuscript.

## Declaration of generative AI and AI-assisted technologies in the writing process

During the preparation of this work, the authors used Gemini service (https://gemini.google.com/app) and Bing Copilot (https://copilot.microsoft.com) in order to improve the readability and language of the manuscript. These services were not used for analyzing and drawing insights from data. After using the services, the authors carefully reviewed and edited the content as needed and take full responsibility for the content of the published article.

## Supporting Material

Document S1. Figures S1–S12, Tables S1–S6, and Supplemental Results

Document S2. Article plus Supporting Material
