# Supplementary material for: Structural and functional characterization of the Pro64Ser leptin mutant: Implications for congenital leptin deficiency
Source: Biophys J. 2025 Aug 28;124(22):4018–33. doi: 10.1016/j.bpj.2025.08.026 (PMC12709403; doi:10.1016/j.bpj.2025.08.026)
Supplement: Document S1. Figures S1–S12, Tables S1–S6, and Supplemental Results [file mmc1.pdf]

Biophysical Journal, Volume *124*

Supplemental information

Structural and functional characterization of the Pro64Ser leptin mutant: Implications for congenital leptin deficiency

Bao Quoc Ngo, Outi Lampela, and André H. Juffer

Supplemental information

1. SUPPLEMENTAL FIGURES

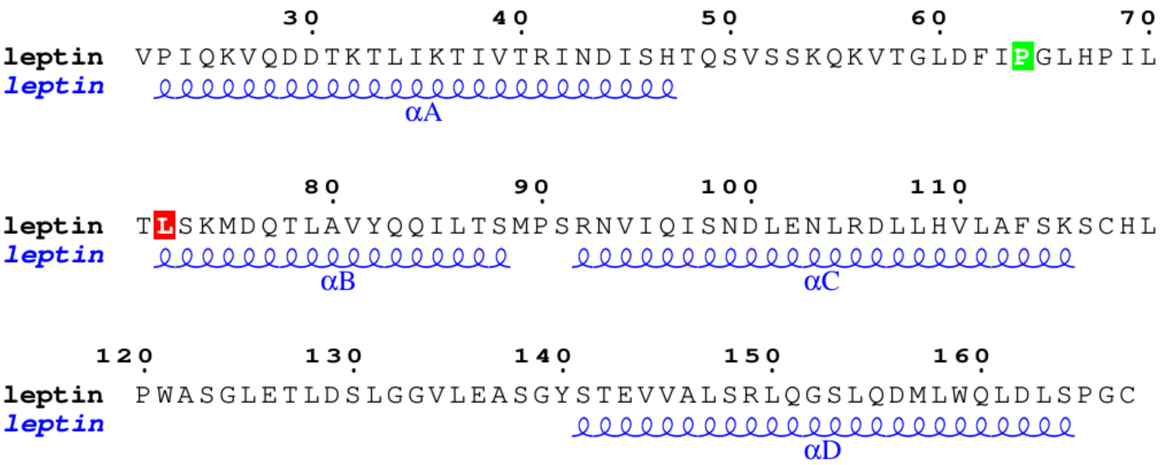

Figure S1. Amino acid sequence and numbering of the leptin protein. The white character with a green background indicates the position of the amino acid Pro64 in the sequence. The white character with a red background denote the position of the amino acid Leu72. The figure was produced using the ESPript 3 server (https://espript.ibcp.fr/ESPript/cgi-bin/ESPript.cgi) (1).

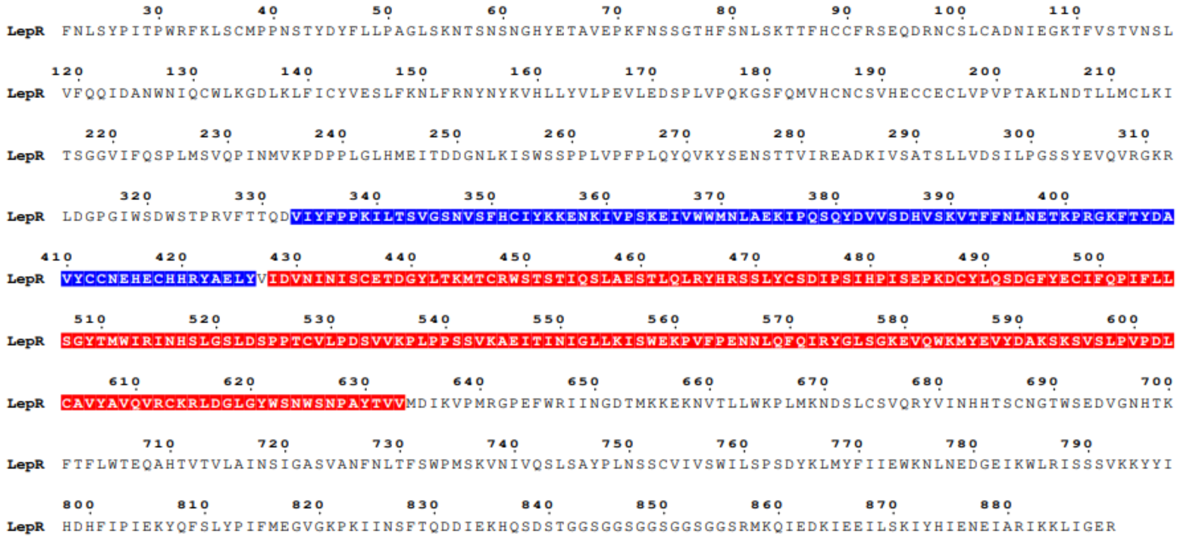

Figure S2. Amino acid sequence and numbering of the LepR. The white characters with a blue background represent the amino acids that are part of the LepR IgD (residues Val333 to Tyr426). The white characters with a red background denote the amino acids that belong to the CRH2 domain (residues Ile428 to Val633) of the LepR. The figure was produced using the ESPript 3 server (https://espript.ibcp.fr/ESPript/cgi-bin/ESPript.cgi) (1).

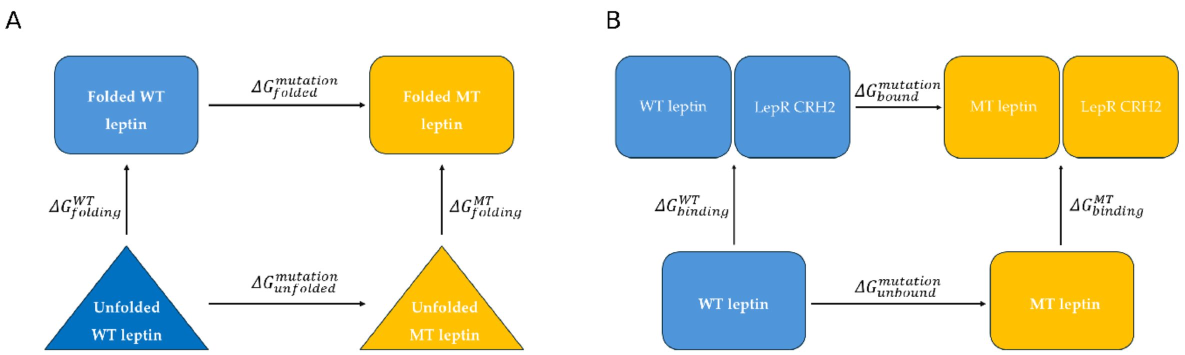

Figure S3. Thermodynamic cycles for computing (A) leptin folding free energy change ($\Delta\Delta G_{folding}^{mutation}$) and (B) leptin binding free energy change for the LepR CRH2 domain ($\Delta\Delta G_{binding}^{mutation}$) upon the mutation.

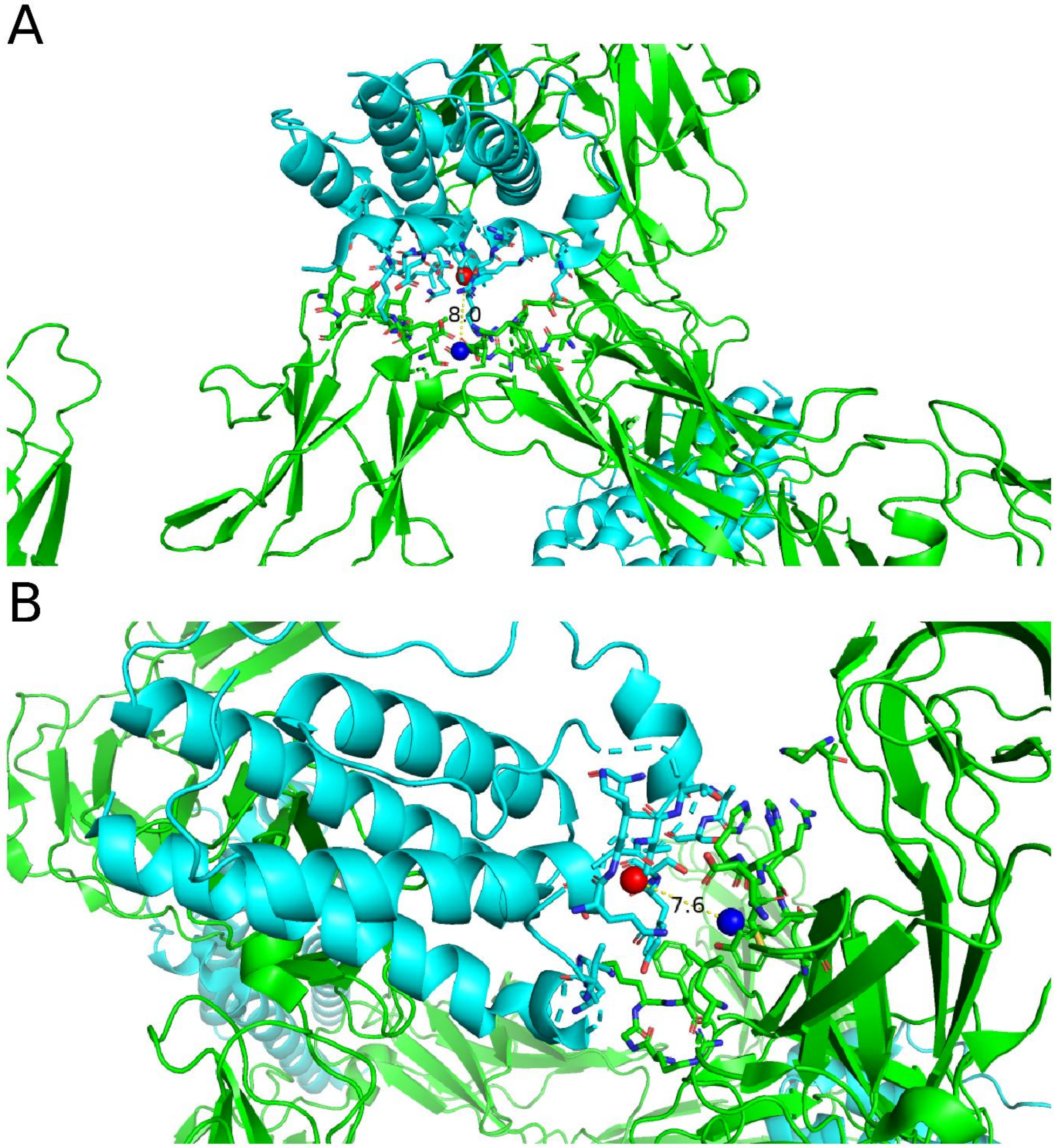

Figure S4. Representation of the distance between the centers of geometry of the leptin interface residues (depicted as red spheres) and the LepR interface residues (depicted as blue spheres) in the 3:3 human leptin-LepR signaling complexes (PDB ID: 8AVF) (2). (A) Geometric center distance between leptin and LepR CRH2 (~8.0Å or 0.8 nm). (B) Geometric center distance between leptin and LepR IgD (~7.6Å or 0.76 nm). Leptin and LepR molecules are colored cyan and green, respectively. The interface residues of leptin and LepR CRH2 are illustrated as sticks and are colored cyan and green, respectively.

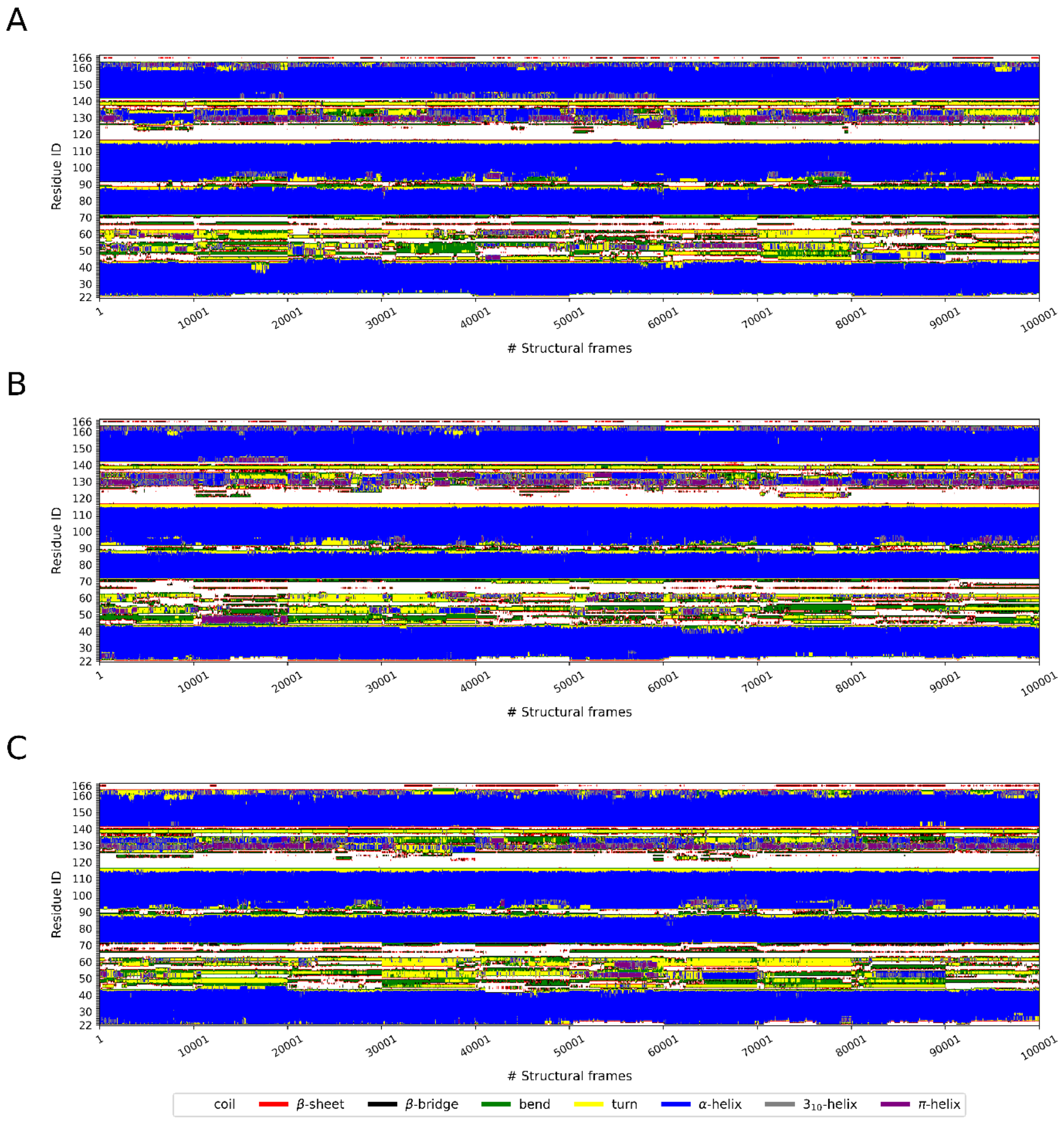

Figure S5. The secondary structure profile of the WT (A), Pro64Ser (B) and Leu72Ser (C) leptins throughout every structural frame of their respective atomistic concatenated trajectories. The color code used to describe the secondary structures is as follows: random coil (white), β-sheet (red), β-bridge (black), bend (green), turn (yellow), α-helix (blue), 3_{10}-helix (gray) and π-helix (purple).

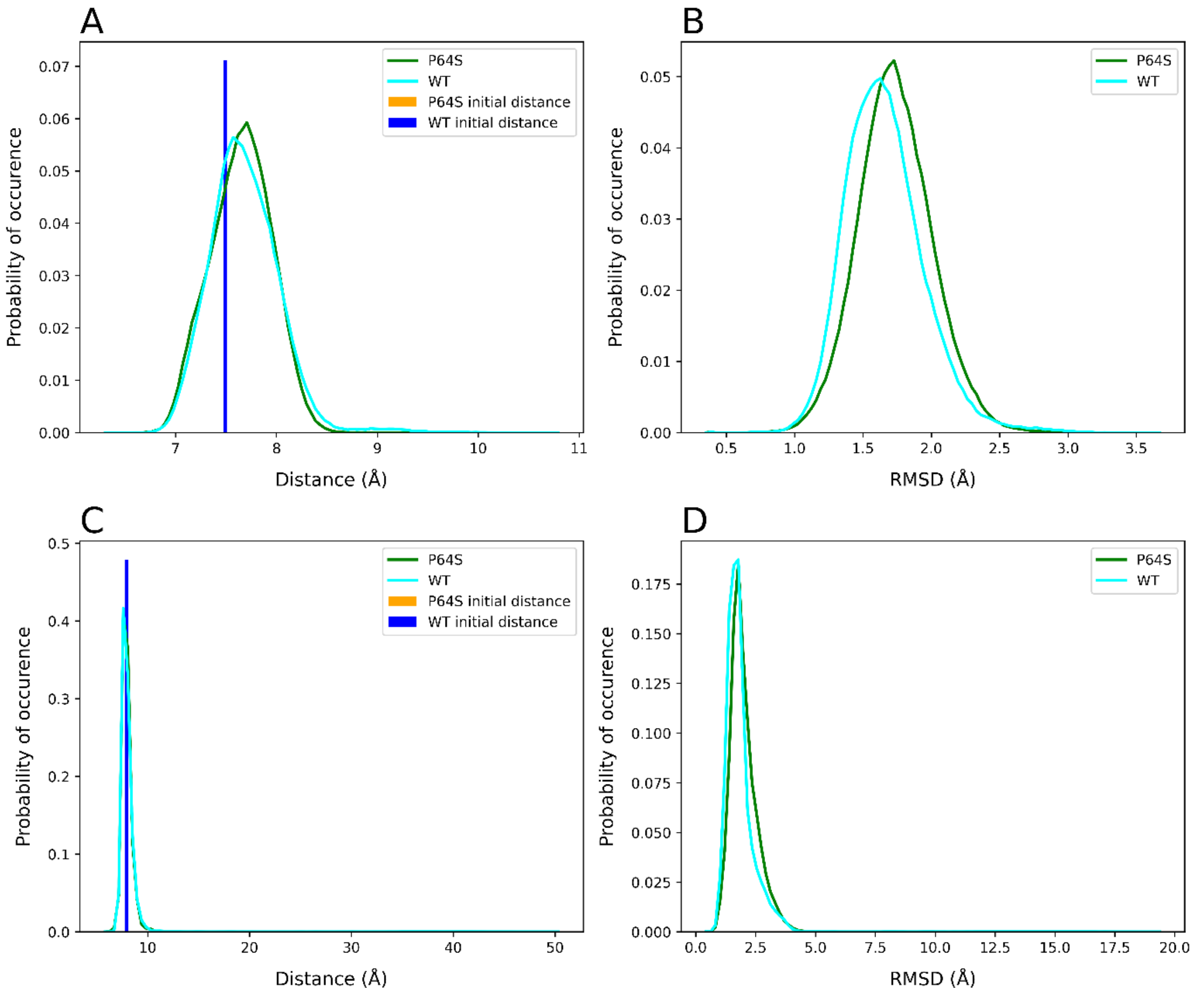

Figure S6. Probability distributions of center-of-geometry binding interfacial distances and RMSD values derived from multiple ATMD simulations of the 3:3 WT and MT Pro64Ser leptin-LepR assemblies. Distribution of geometric center distances between the binding interfaces of the WT/MT leptin and the LepR CRH2 (A) and IgD (C). Distribution of the sampled RMSD values for the leptin-CRH2 (B) and leptin-IgD (D) monomeric complexes (relative to the corresponding complexes in the refined structure; refer to Supplemental Results for further details). The distribution curves for the WT and MT leptin-LepR complexes are depicted in cyan and green, respectively. In subfigures A and C, the initial geometric center distances for the WT and MT complexes, averaged from the three leptin-CRH2/IgD interfacial distances in the refined 3:3 assembly, are represented as bars and colored blue and orange, respectively.

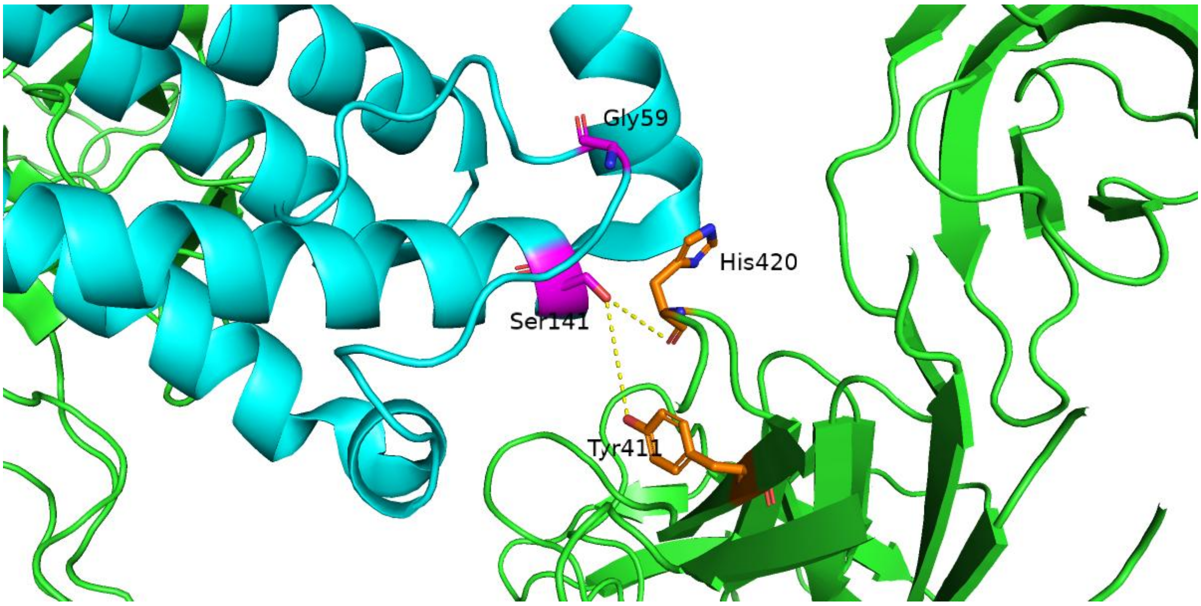

Figure S7. Spatial positioning of residues Gly59 and Ser141 (displayed as magenta sticks) on the WT leptin structure (shown in cyan), relative to the IgD of the LepR (shown in green). The Gly59Ser substitution (3) and the Ser141Cys substitution (4) have been shown to impair the capability of leptin to stimulate the LepR intracellular signaling. Potential hydrogen bonds between Ser141 and the LepR residues His420 and Tyr411 (depicted as orange sticks) are indicated by yellow dashed lines. The figure was generated using PyMol Molecular Graphics System (Open-Source, Schrödinger, LLC).

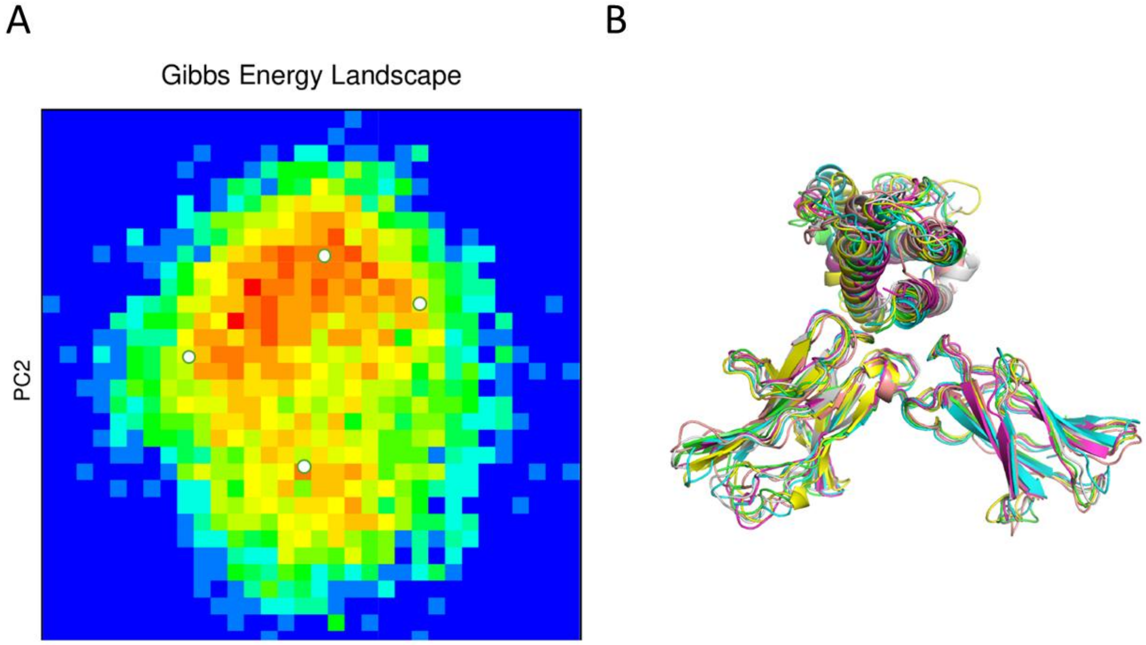

Figure S8. Structural characterization of the WT leptin–LepR CRH2 complex sampled during equilibrium simulation used in binding free energy computation workflow. (A) Conformational landscape of the leptin–CRH2 complex, constructed via principal component analysis of the simulation trajectory. (B) Representative conformations of the leptin–LepR CRH2 complex extracted from distinct regions of the conformational landscape (marked in white spot). These structures were aligned using PyMOL software to assess structural variability. The alignment reveals no significant rearrangement in the binding interface, indicating a stable binding mode throughout the simulation. Subfigures A and B were generated using the GROMACS analysis toolkit and the PyMOL Molecular Graphics System (Open-Source, Schrödinger, LLC), respectively.

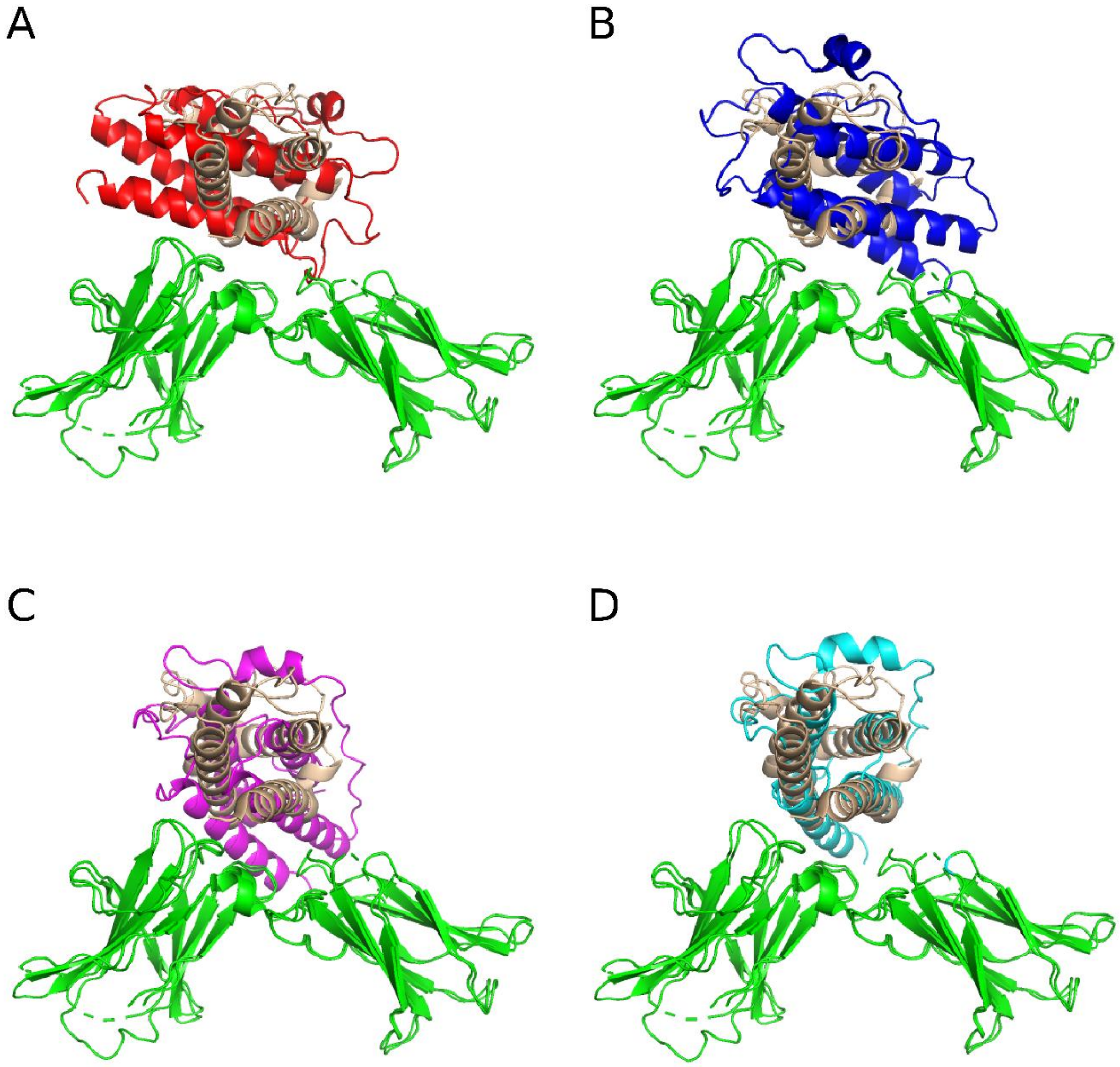

Figure S9. Predicted binding conformations of leptin with the isolated LepR CRH2 domain, generated via HADDOCK2.4 docking simulations (5,6).

Panels A-C illustrate the top three binding conformations of modeled leptin (colored red, blue, and purple, respectively) docked to the isolated CRH2 domain (PDB ID: 3V6O (7); shown in green). Note that the binding conformations were ranked by complex stability as determined by HADDOCK score. The HADDOCK score is a weighted linear combination of various energy terms, including van der Waals, electrostatic, desolvation, and restraint violation energies (8). The corresponding HADDOCK scores (arbitrary units) for the leptin's binding conformations in panels A, B, and C are −76.6 ± 9.5, −66.0 ± 9.7, and −61.2 ± 9.5, respectively.

Panel D presents the leptin-CRH2 binding conformation that is the most structurally similar to that observed in the 3:3 leptin–LepR complex (PDB ID: 8AVF) (2), as predicted by HADDOCK. This conformation, which was ranked seventh in terms of stability, has a HADDOCK score of −50.9 ± 7.5. In all panels, the CRH2 domain is depicted in green, and the semi-transparent (tinted) leptin represents its conformation within the 3:3 assembly for structural comparison.

The figure was generated using PyMol Molecular Graphics System (Open-Source, Schrödinger, LLC).

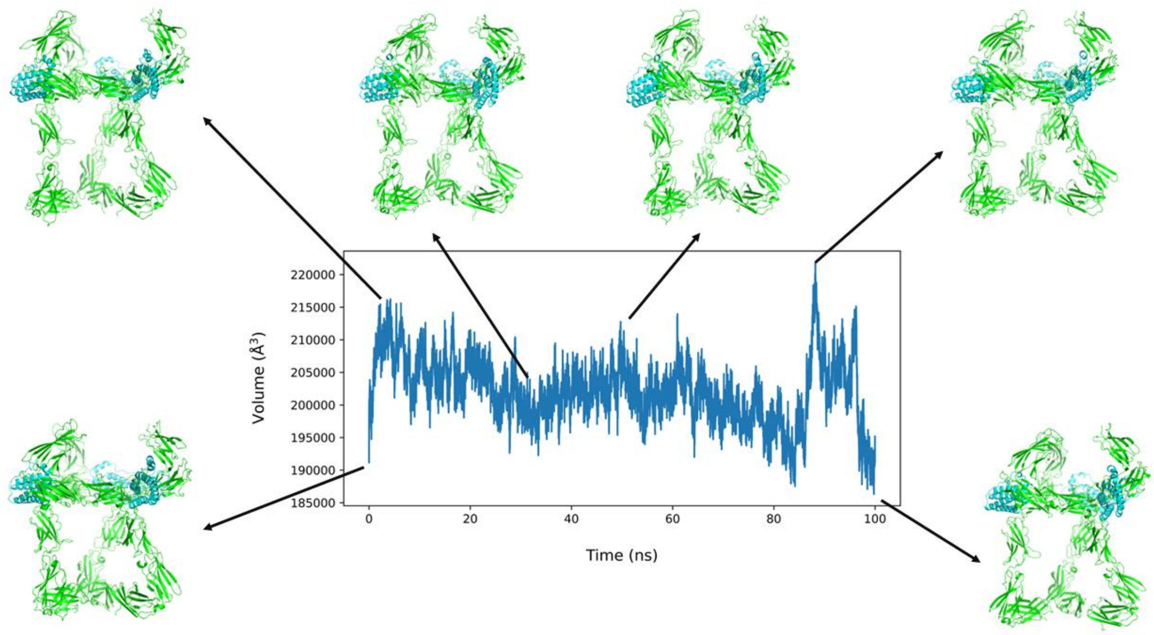

Figure S10. Evolution of the volume enclosed by the FNIII domains of the three LepR monomers, along with the selected complex structures at distinct time steps along the trajectory generated from the 3:3 leptin-LepR refinement simulation.

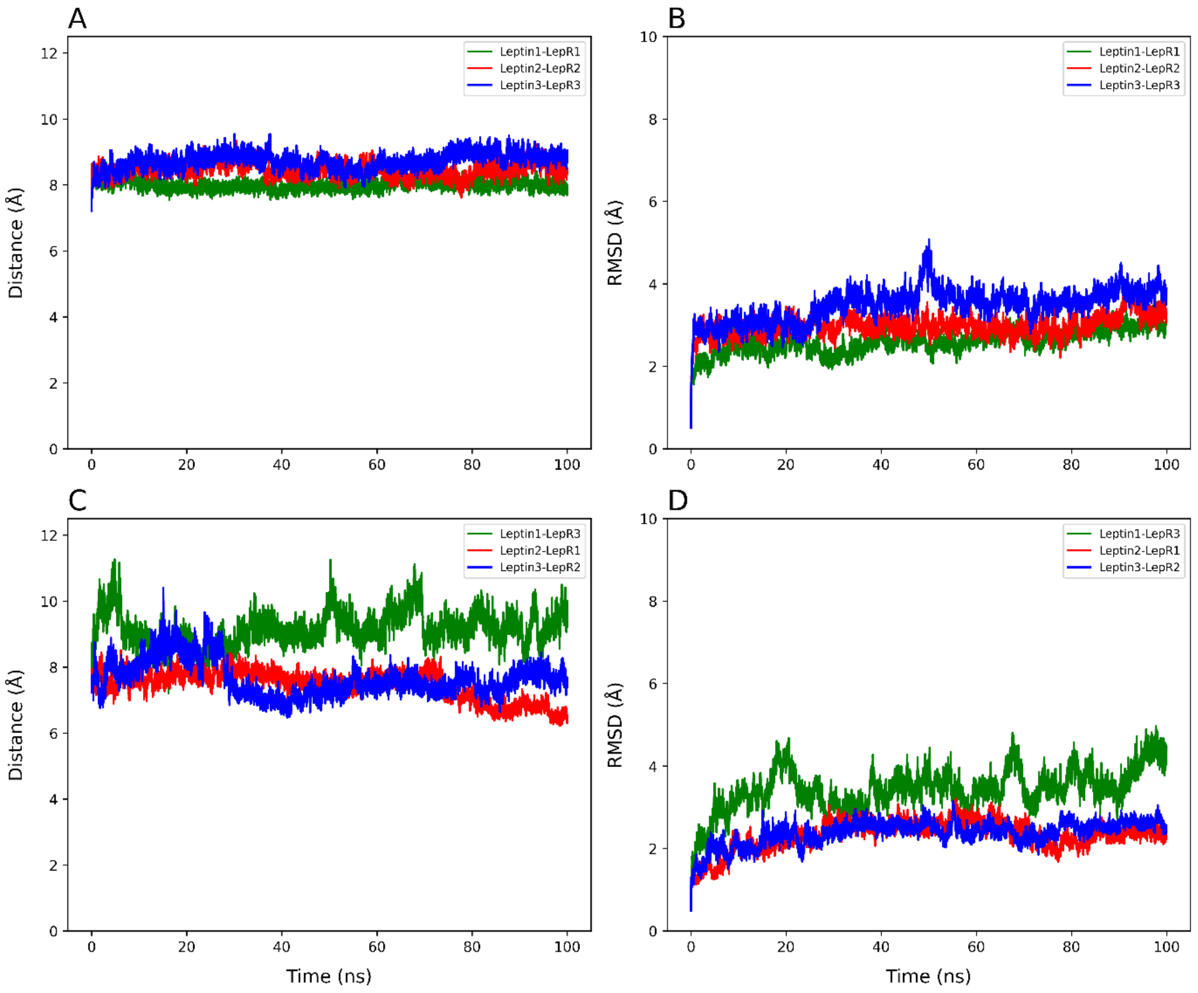

Figure S11. Evolution of the geometric center distance between leptin and the LepR CRH2's binding interfaces (A) and between leptin and the LepR IgD's binding interfaces (C) throughout the 3:3 leptin-LepR refinement simulation. RMSD evolution of the monomeric leptin-LepR CRH2 complexes (B) and leptin-LepR IgD complexes (D). The geometric center distance and RMSD evolution curves that involve leptin1, leptin2 and leptin3 are colored green, red and blue, respectively. Leptin1, leptin2 and leptin3 correspond to the molecules designated as chain A, chain C and chain E in the 3:3 human leptin-LepR assembly (PDB ID: 8AVF) (2). Similarly, LepR1, LepR2 and LepR3 correspond to the molecules denoted as chain B, chain D and chain F in the homotrimeric structure.

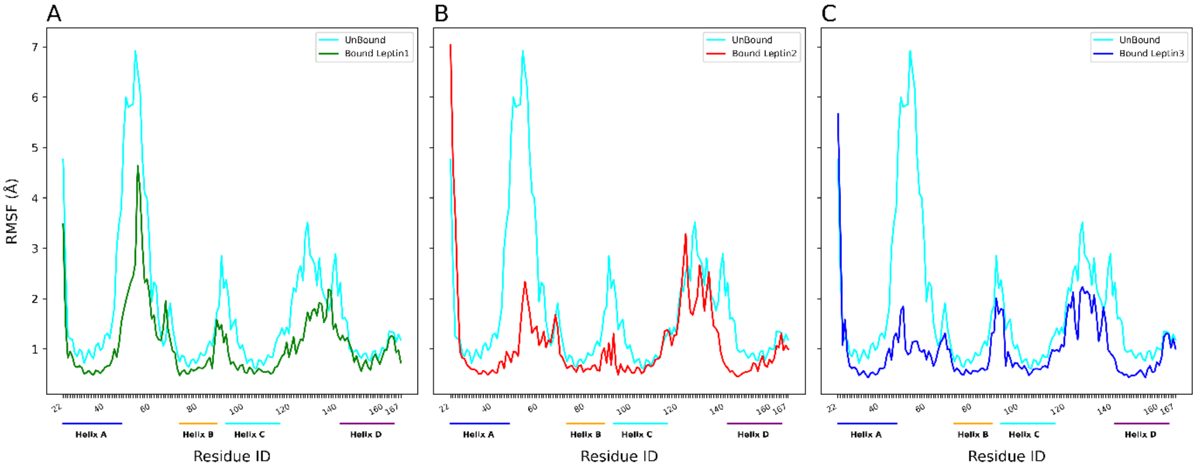

Figure S12. The Root Mean Square Fluctuation (RMSF) profiles of the Cα atoms of leptin in its unbound state (cyan curve) and bound states (green, red and blue curves). The RMSF profile for the unbound leptin was derived from the concatenated trajectory of the WT leptin's 10 independent ATMD simulations (for details see main text section 2.3.1). The RMSF profile for each bound leptin molecule was generated from the 3:3 human leptin-LepR complex refinement simulation (refer section 2.4.1 for details). Leptin1 (green curve and subfigure A), leptin2 (red curve and subfigure B), and leptin3 (blue curve and subfigure C) correspond to chains A, C and E in the 3:3 human leptin-LepR ectodomain (PDB ID: 8AVF) (2).

2. SUPPLEMENTAL TABLES

Table S1. Statistical evaluation of the distribution curves presented in Fig. 3 in the main text. WT/P64S/L72S mean distance represents the mean distance between the side chain centers of geometry of the residue 72 and another inspected residue throughout the simulations. WT/P64S/L72S initial distance refers to the average side chain center of geometry distance between the residue 72 and inspected residue, measured across the first structural frames in each 10 independent WT/MT trajectories. WT/P64S/L72S variance denotes the variance of the sampled distance from the WT/MT leptin simulations. WT/P64S/L72S initial distance population indicates the proportion of structural frames from the WT/MT simulations, where the distance between residue 72 and the examined residue falls within 5% of their initial distance. Note that the residues that are in close proximity to the WT Leu72 are denoted by an asterisk (*) adjacent to their residue IDs.

Residue ID	WT mean distance (Å)	P64S mean distance (Å)	L72S mean distance (Å)	WT initial distance (Å)	P64S initial distance (Å)	L72S initial distance (Å)	WT variance ($Å^2$)	P64S variance ($Å^2$)	L72S variance ($Å^2$)	WT initial distance population	P64S initial distance population	L72S initial distance population
Thr31*	8.81	8.72	9.67	8.96	8.94	9.42	0.28	0.27	0.80	0.58	0.56	0.39
Ile35	12.3	12.21	12.82	12.47	12.35	12.37	0.36	0.4	0.85	0.69	0.70	0.44
Ile38	16.57	16.65	16.80	16.64	16.64	16.73	0.23	0.23	0.56	0.92	0.92	0.76
Met75*	5.72	5.75	6.00	5.83	5.78	5.35	0.14	0.15	0.80	0.55	0.59	0.31
Thr78*	11.8	11.76	11.28	12.03	11.9	11.34	0.12	0.12	0.12	0.87	0.90	0.90
Leu79*	10.79	10.84	10.48	10.65	10.74	10.20	0.13	0.14	0.21	0.85	0.85	0.68
Tyr82	16.75	16.98	16.12	16.51	16.48	15.94	0.19	0.25	0.31	0.91	0.75	0.85
Ile97	21.43	21.61	21.43	20.93	20.9	20.71	0.25	0.32	0.48	0.86	0.74	0.68
Leu101	15.52	15.64	15.39	15.29	15.17	15.10	0.17	0.18	0.29	0.90	0.76	0.81
Leu104*	10.93	11.07	11.13	10.86	10.93	10.84	0.20	0.20	0.46	0.81	0.79	0.56
Leu108*	5.45	5.52	5.32	5.33	5.37	5.36	0.10	0.11	0.29	0.60	0.60	0.42
Ala146	23.55	23.63	23.08	23.33	23.4	22.83	0.27	0.29	0.60	0.96	0.95	0.85
Leu150	17.55	17.6	17.31	17.36	17.4	16.91	0.21	0.25	0.49	0.93	0.91	0.73
Ser153	15.03	15.18	14.48	14.86	14.71	14.34	0.30	0.29	0.73	0.84	0.70	0.59
Leu154	14.63	14.67	14.69	13.99	13.9	14.01	0.27	0.29	0.67	0.56	0.47	0.48
Met157*	9.41	9.45	9.33	9.51	9.36	9.12	0.34	0.34	1.54	0.61	0.62	0.30

Table S2. Hydrogen bond interactions between selected amino acids from the AB loop and their surrounding residues within the WT leptin structure, as identified using the hydrogen bond analysis scheme developed by Ngo & Juffer (2024). The interactions listed in this table were observed across the multiple ATMD simulations of the unbound WT leptin.

The "Residue ID" column specifies the amino acids selected for hydrogen bond analysis. The "Interaction amino acid pairs" column enumerates the pairs of amino acids that formed hydrogen bonds during the simulations. An asterisk (*) next to a pair indicates that the interaction involves an amino acid from one of the four major α-helices in the leptin structure. The "Interaction atom pairs" column details the specific atom pairs involved in these hydrogen bonds.

The "Number of frames" column indicates the number of frames (out of approximately 100,000 total frames), in which the specified atom pairs were observed to form hydrogen bonds. The "Probability" column represents the likelihood of a hydrogen bond formation, as calculated by dividing the number of frames by the total number of frames in the concatenated trajectory. Only atom pairs with a hydrogen bonding probability greater than 0.02 are included in this table. Full data can be accessed via the provided link (https://github.com/quocbaongo/Leptin_LepR_Research/tree/main/MultiSimulations_Result_and_Analysis/Multi_WT_MT_leptin_Simulations/Python_Plotting_Scripts/HbondNetwork/WTRes_HbondNetwork).

Additionally, the total number of hydrogen bonds were detected between any selected amino acid within the AB loop and amino acids from the α-helices, AB loop itself, and CD loop were calculated based on all detected atom pairs forming hydrogen bonds throughout the simulations.

Residue ID	Interaction amino acid pairs	Interaction atom pairs	Number of frames	Probability	Backbone (BB) - side chain (SC)
Ile45	Ser50-Ile45	OG Ser50······ O Ile45	2139	0.021	SC-BB

Number of detected H-bond between Ile45 and **major α-helices**: 1357

Number of detected H-bond between Ile45 and **AB loop**: 3871

Number of detected H-bond between Ile45 and **CD loop**: 0

Residue ID	Interaction amino acid pairs	Interaction atom pairs	Number of frames	Probability	Backbone (BB) - side chain (SC)
Ser46	Ser46-Asp44*	OG Ser46······ OD2 Asp44	47067	0.471	SC-SC
	Ser46-Ile42*	OG Ser46······ O Ile42	5623	0.056	SC-BB
	Asn93-Ser46*	ND2 Asn93······ O Ser46	3218	0.032	SC-BB

Number of detected H-bond between Ser46 and **major α-helices**: 60298

Number of detected H-bond between Ser46 and **AB loop**: 1806

Number of detected H-bond between Ser46 and **CD loop**: 0

Residue ID	Interaction amino acid pairs	Interaction atom pairs	Number of frames	Probability	Backbone (BB) - side chain (SC)
His47	His47-Arg41*	NE2 His47······ O Arg41	9373	0.094	SC-BB
	His47-Ile42*	NE2 His47······ O Ile42	4633	0.046	SC-BB
	Ser50-His47	OG Ser50······ ND1 His47	3246	0.032	SC-SC
	His47-Val57	NE2 His47······ O Val57	2315	0.023	SC-BB
	His47-Glu143*	NE2 His47······ OE1 Glu143	2170	0.022	SC-SC
	Asn93-His47*	ND2 Asn93······ ND1 His47	2007	0.02	SC-SC

Number of detected H-bond between His47 and **major α-helices**: 19736

Number of detected H-bond between His47 and **AB loop**: 9415

Number of detected H-bond between His47 and **CD loop**: 0

Residue ID	Interaction amino acid pairs	Interaction atom pairs	Number of frames	Probability	Backbone (BB) - side chain (SC)
Thr48	Asn93-Thr48*	ND2 Asn93⋯⋯ OG1 Thr48	5392	0.054	SC-SC
	Thr48-Asp44*	OG1 Thr48⋯⋯ O Asp44	2404	0.024	SC-BB

Number of detected H-bond between Thr48 and **major α-helices**: 11060

Number of detected H-bond between Thr48 and **AB loop**: 4777

Number of detected H-bond between Thr48 and **CD loop**: 1

Residue ID	Interaction amino acid pairs	Interaction atom pairs	Number of frames	Probability	Backbone (BB) - side chain (SC)
Gln49	Gln49-Glu143*	NE2 Gln49⋯⋯ OE1 Glu143	3232	0.032	SC-SC
	Ser53-Gln49	OG Ser53⋯⋯ O Gln49	2182	0.022	SC-BB

Number of detected H-bond between Gln49 and **major α-helices**: 7192

Number of detected H-bond between Gln49 and **AB loop**: 8539

Number of detected H-bond between Gln49 and **CD loop**: 1268

Residue ID	Interaction amino acid pairs	Interaction atom pairs	Number of frames	Probability	Backbone (BB) - side chain (SC)
Ser50	Ser53-Ser50	OG Ser53⋯⋯ OG Ser50	10010	0.1	SC-SC
	Ser50-Asp44*	OG Ser50⋯⋯ O Asp44	6431	0.064	SC-BB
	Ser91-Ser50	OG Ser91⋯⋯ OG Ser50	5211	0.052	SC-SC
	Gln55-Ser50	NE2 Gln55⋯⋯ O Ser50	4079	0.041	SC-BB
	Ser50-His47*	OG Ser50⋯⋯ ND1 His47	3246	0.032	SC-SC
	Ser50-Lys56	OG Ser50⋯⋯ O Lys56	2728	0.027	SC-BB
	Ser50-Glu143*	OG Ser50⋯⋯ O Glu143	2329	0.023	SC-BB
	Ser50-Ile45*	OG Ser50⋯⋯ O Ile45	2139	0.021	SC-BB

Number of detected H-bond between Ser50 and **major α-helices**: 16070

Number of detected H-bond between Ser50 and **AB loop**: 21745

Number of detected H-bond between Ser50 and **CD loop**: 0

Residue ID	Interaction amino acid pairs	Interaction atom pairs	Number of frames	Probability	Backbone (BB) - side chain (SC)
Ser52	Ser52-Asp44*	OG Ser52⋯⋯ OD2 Asp44	3989	0.04	SC-SC
	Ser52-Lys56	OG Ser52⋯⋯ O Lys56	3191	0.032	SC-BB
	Ser52-Asn43*	OG Ser52⋯⋯ O Asn43	3159	0.032	SC-BB
	Gln55-Ser52	NE2 Gln55⋯⋯ O Ser52	2602	0.026	SC-BB

Number of detected H-bond between Ser52 and **major α-helices**: 10991

Number of detected H-bond between Ser52 and **AB loop**: 10330

Number of detected H-bond between Ser52 and **CD loop**: 1633

Residue ID	Interaction amino acid pairs	Interaction atom pairs	Number of frames	Probability	Backbone (BB) - side chain (SC)
Ser53	Ser53-Ser50	OG Ser53⋯⋯ OG Ser50	10010	0.01	SC-SC
	Ser53-Gln49	OG Ser53⋯⋯ O Gln49	2182	0.022	SC-BB
Number of detected H-bond between Ser53 and **major α-helices**: 6504					
Number of detected H-bond between Ser53 and **AB loop**: 14689					
Number of detected H-bond between Ser53 and **CD loop**: 118					

Residue ID	Interaction amino acid pairs	Interaction atom pairs	Number of frames	Probability	Backbone (BB) - side chain (SC)
Lys54	Lys54-Glu143*	NZ Lys54⋯⋯ OE2 Glu143	4496	0.045	SC-SC
Number of detected H-bond between Lys54 and **major α-helices**: 6001					
Number of detected H-bond between Lys54 and **AB loop**: 6259					
Number of detected H-bond between Lys54 and **CD loop**: 2780					

Residue ID	Interaction amino acid pairs	Interaction atom pairs	Number of frames	Probability	Backbone (BB) - side chain (SC)
Gln55	Gln55-Ser50	NE2 Gln55⋯⋯ O Ser50	4079	0.041	SC-BB
	Gln55-Ser52	NE2 Gln55⋯⋯ O Ser52	2602	0.026	SC-BB
Number of detected H-bond between Gln55 and **major α-helices**: 4055					
Number of detected H-bond between Gln55 and **AB loop**: 10138					
Number of detected H-bond between Gln55 and **CD loop**: 188					

Residue ID	Interaction amino acid pairs	Interaction atom pairs	Number of frames	Probability	Backbone (BB) - side chain (SC)
Lys56	Ser52-Lys56	OG Ser52⋯⋯ O Lys56	3191	0.032	SC-BB
	Lys56-Asp61	NZ Lys56⋯⋯ OD2 Asp61	2788	0.028	SC-SC
	Ser50-Lys56	OG Ser50⋯⋯ O Lys56	2728	0.027	SC-BB
	Lys56-Ser138	NZ Lys56⋯⋯ O Ser138	2727	0.027	SC-BB
	Tyr140-Lys56	OH Tyr140⋯⋯ O Lys56	2134	0.021	SC-BB
Number of detected H-bond between Lys56 and **major α-helices**: 2230					
Number of detected H-bond between Lys56 and **AB loop**: 11788					
Number of detected H-bond between Lys56 and **CD loop**: 5745					

Residue ID	Interaction amino acid pairs	Interaction atom pairs	Number of frames	Probability	Backbone (BB) - side chain (SC)
Val57	His47-Val57*	NE2 His47⋯⋯ O Val57	2315	0.023	SC-BB
Number of detected H-bond between Val57 and **major α-helices**: 2524					
Number of detected H-bond between Val57 and **AB loop**: 383					
Number of detected H-bond between Val57 and **CD loop**: 118					

Residue ID	Interaction amino acid pairs	Interaction atom pairs	Number of frames	Probability	Backbone (BB) - side chain (SC)
Thr58	Tyr140-Thr58	OH Tyr140…… O Thr58	32777	0.328	SC-BB
	Thr58-Ser148*	OG1 Thr58…… OG Ser148	3693	0.037	SC-SC

Number of detected H-bond between Thr58 and **major α-helices**: 6617

Number of detected H-bond between Thr58 and **AB loop**: 3052

Number of detected H-bond between Thr58 and **CD loop**: 33351

Residue ID	Interaction amino acid pairs	Interaction atom pairs	Number of frames	Probability	Backbone (BB) - side chain (SC)
Pro64	Arg149-Pro64*	NH1 Arg149…… O Pro64	61193	0.612	SC-BB

Number of detected H-bond between Pro64 and **major α-helices**: 61194

Number of detected H-bond between Pro64 and **AB loop**: 0

Number of detected H-bond between Pro64 and **CD loop**: 0

Table S3. Hydrogen bond interactions between selected amino acids from the AB loop and their surrounding residues within the MT Pro64Ser leptin structure, detected using the analysis scheme developed by Ngo & Juffer (2024). The listed interactions were identified over the multiple ATMD simulations of the unbound MT Pro64Ser leptin. Full data can be accessed via the provided link (https://github.com/quocbaongo/Leptin_LepR_Research/tree/main/MultiSimulations_Result_and_Analysis/Multi_WT_MT_leptin_Simulations/Python_Plotting_Scripts/HbondNetwork/P64SRes_HbondNetwork). Other details are similar as Table S2.

Residue ID	Interaction amino acid pairs	Interaction atom pairs	Number of frames	Probability	Backbone (BB) - side chain (SC)
Ile45	Thr48-Ile45	OG1 Thr48⋯⋯ O Ile45	6079	0.061	SC-BB

Number of detected H-bond between Ile45 and **major α-helices**: 47

Number of detected H-bond between Ile45 and **AB loop**: 8848

Number of detected H-bond between Ile45 and **CD loop**: 0

Residue ID	Interaction amino acid pairs	Interaction atom pairs	Number of frames	Probability	Backbone (BB) - side chain (SC)
Ser46	Ser46-Asp44*	OG Ser46⋯⋯ OD1 Asp44	63308	0.633	SC-SC
	Asn93-Ser46*	ND2 Asn93⋯⋯ OG Ser46	4217	0.042	SC-SC

Number of detected H-bond between Ser46 and **major α-helices**: 69374

Number of detected H-bond between Ser46 and **AB loop**: 1994

Number of detected H-bond between Ser46 and **CD loop**: 0

Residue ID	Interaction amino acid pairs	Interaction atom pairs	Number of frames	Probability	Backbone (BB) - side chain (SC)
His47	His47-Arg41*	NE2 His47⋯⋯ O Arg41	14967	0.15	SC-BB
	His47-Glu143*	NE2 His47⋯⋯ OE1 Glu143	2549	0.025	SC-SC
	Asn93-His47*	ND2 Asn93⋯⋯ ND1 His47	2337	0.023	SC-SC
	Ser50-His47	OG Ser50⋯⋯ ND1 His47	2142	0.021	SC-SC

Number of detected H-bond between His47 and **major α-helices**: 21304

Number of detected H-bond between His47 and **AB loop**: 5917

Number of detected H-bond between His47 and **CD loop**: 0

Residue ID	Interaction amino acid pairs	Interaction atom pairs	Number of frames	Probability	Backbone (BB) - side chain (SC)
Thr48	Asn93-Thr48*	ND2 Asn93⋯⋯ OG1 Thr48	6821	0.068	SC-SC
	Thr48-Ile45*	OG1 Thr48⋯⋯ O Ile45	6079	0.061	SC-BB
	Thr48-Asn93*	OG1 Thr48⋯⋯ OD1 Asn93	4986	0.05	SC-SC
	Thr48-Gln49	OG1 Thr48⋯⋯ OE1 Gln49	2165	0.022	SC-SC

Number of detected H-bond between Thr48 and **major α-helices**: 22954

Number of detected H-bond between Thr48 and **AB loop**: 4299

Number of detected H-bond between Thr48 and **CD loop**: 0

Residue ID	Interaction amino acid pairs	Interaction atom pairs	Number of frames	Probability	Backbone (BB) - side chain (SC)
Gln49	Gln49-Glu143*	NE2 Gln49…… OE1 Glu143	8615	0.086	SC-SC
	Ser141-Gln49*	OG Ser141…… OE1 Gln49	3562	0.036	SC-SC
	Thr48-Gln49	OG1 Thr48…… OE1 Gln49	2165	0.022	SC-SC

Number of detected H-bond between Gln49 and **major α-helices**: 21904
Number of detected H-bond between Gln49 and **AB loop**: 6817
Number of detected H-bond between Gln49 and **CD loop**: 1759

Residue ID	Interaction amino acid pairs	Interaction atom pairs	Number of frames	Probability	Backbone (BB) - side chain (SC)
Ser50	Ser50-Asp44*	OG Ser50…… O Asp44	8238	0.082	SC-BB
	Ser53-Ser50	OG Ser53…… O Ser50	6067	0.061	SC-BB
	Ser50-Gln55	OG Ser50…… O Gln55	3999	0.04	SC-BB
	Ser91-Ser50	OG Ser91…… OG Ser50	3714	0.037	SC-SC
	Asn93-Ser50*	ND2 Asn93…… OG Ser50	3185	0.032	SC-SC
	Ser50-His47*	OG Ser50…… ND1 His47	2142	0.021	SC-SC

Number of detected H-bond between Ser50 and **major α-helices**: 17825
Number of detected H-bond between Ser50 and **AB loop**: 12730
Number of detected H-bond between Ser50 and **CD loop**: 23

Residue ID	Interaction amino acid pairs	Interaction atom pairs	Number of frames	Probability	Backbone (BB) - side chain (SC)
Val51	Ser141-Val51*	OG Ser141…… O Val51	3632	0.036	SC-BB

Number of detected H-bond between Val51 and **major α-helices**: 3633
Number of detected H-bond between Val51 and **AB loop**: 566
Number of detected H-bond between Val51 and **CD loop**: 14

Residue ID	Interaction amino acid pairs	Interaction atom pairs	Number of frames	Probability	Backbone (BB) - side chain (SC)
Ser52	Ser52-Lys56	OG Ser52…… O Lys56	12109	0.121	SC-BB
	Ser52-Asn43*	OG Ser52…… OD1 Asn43	6274	0.063	SC-SC
	Ser52-Asp44*	OG Ser52…… O Asp44	2730	0.027	SC-BB

Number of detected H-bond between Ser52 and **major α-helices**: 11440
Number of detected H-bond between Ser52 and **AB loop**: 16856
Number of detected H-bond between Ser52 and **CD loop**: 1080

Residue ID	Interaction amino acid pairs	Interaction atom pairs	Number of frames	Probability	Backbone (BB) - side chain (SC)
Ser53	Ser53-Glu143*	OG Ser53⋯⋯ OE1 Glu143	6625	0.066	SC-SC
	Ser53-Ser50	OG Ser53⋯⋯ O Ser50	6067	0.061	SC-BB

Number of detected H-bond between Ser53 and **major α-helices**: 8564
Number of detected H-bond between Ser53 and **AB loop**: 9039
Number of detected H-bond between Ser53 and **CD loop**: 214

Residue ID	Interaction amino acid pairs	Interaction atom pairs	Number of frames	Probability	Backbone (BB) - side chain (SC)
Lys54	Lys54-Gly139	NZ Lys54⋯⋯ O Gly139	3074	0.031	SC-BB
	Asn43-Lys54*	ND2 Asn43⋯⋯ O Lys54	2550	0.025	SC-BB

Number of detected H-bond between Lys54 and **major α-helices**: 3377
Number of detected H-bond between Lys54 and **AB loop**: 4691
Number of detected H-bond between Lys54 and **CD loop**: 6256

Residue ID	Interaction amino acid pairs	Interaction atom pairs	Number of frames	Probability	Backbone (BB) - side chain (SC)
Gln55	Ser50-Gln55	OG Ser50⋯⋯ O Gln55	3999	0.04	SC-BB

Number of detected H-bond between Gln55 and **major α-helices**: 8554
Number of detected H-bond between Gln55 and **AB loop**: 8461
Number of detected H-bond between Gln55 and **CD loop**: 87

Residue ID	Interaction amino acid pairs	Interaction atom pairs	Number of frames	Probability	Backbone (BB) - side chain (SC)
Lys56	Ser52-Lys56	OG Ser52⋯⋯ O Lys56	12109	0.121	SC-BB
	Ser141-Lys56*	OG Ser141⋯⋯ O Lys56	4107	0.041	SC-BB
	Lys56-Ser138	NZ Lys56⋯⋯ OG Ser138	2905	0.029	SC-SC
	Tyr140-Lys56	OH Tyr140⋯⋯ O Lys56	2307	0.023	SC-BB

Number of detected H-bond between Lys56 and **major α-helices**: 8522
Number of detected H-bond between Lys56 and **AB loop**: 16806
Number of detected H-bond between Lys56 and **CD loop**: 6129

Residue ID	Interaction amino acid pairs	Interaction atom pairs	Number of frames	Probability	Backbone (BB) - side chain (SC)
Thr58	Tyr140-Thr58	OH Tyr140⋯⋯ O Thr58	35165	0.352	SC-BB

Number of detected H-bond between Thr58 and **major α-helices**: 1508
Number of detected H-bond between Thr58 and **AB loop**: 4652
Number of detected H-bond between Thr58 and **CD loop**: 35477

Residue ID	Interaction amino acid pairs	Interaction atom pairs	Number of frames	Probability	Backbone (BB) - side chain (SC)
Ser64	Arg149-Ser64*	NH1 Arg149…… O Ser64	47741	0.477	SC-BB
Number of detected H-bond between Ser64 and **major α-helices**: 49276					
Number of detected H-bond between Ser64 and **AB loop**: 1421					
Number of detected H-bond between Ser64 and **CD loop**: 0					

Table S4. Statistical evaluation of the distribution curves presented in Fig. 8 in the main text. WT/P64S mean distance represents the mean distance between the side chain center-of-geometry of Leu66 and each of the hydrophobic amino acids that are in close proximity to the Leu66 throughout the free WT and Pro64Ser leptins' simulations. WT/P64S initial distance refers to the average side chain center of geometry distance between the Leu66 and inspected residue, measured across the first structural frames in each 10 independent WT/MT trajectories. WT/P64S variance denotes the variance of the sampled distance from the WT/MT leptin simulations. WT/P64S initial distance population indicates the proportion of structural frames from the WT/MT simulations, where the distance between the Leu66 and the examined residue falls within 5% of their initial distance.

**Residue ID**	**WT mean distance (Å)**	**P64S mean distance (Å)**	**WT initial distance (Å)**	**P64S initial distance (Å)**	**WT variance (Å^2)**	**P64S variance (Å^2)**	**WT initial distance population**	**P64S initial distance population**
Ile63	10.96	11.08	9.21	9.2	0.65	1.16	0.06	0.09
Pro/Ser64	9.39	8.94	9.06	8.5	0.31	0.61	0.49	0.36
Leu79	10.69	10.93	10.88	10.8	0.48	1.76	0.59	0.54
Val81	6.3	6.72	5.76	5.73	0.64	2.49	0.32	0.33
Ile85	8.4	9.63	6.44	6.46	1.57	4.23	0.09	0.03
Leu125	9.85	11.88	9.33	9.28	2.62	8.21	0.32	0.16
Leu131	6.47	7.95	6.08	6.05	2.28	10.97	0.28	0.28
Val134	5.99	6.75	5.6	5.52	0.77	4.5	0.36	0.28
Leu135	8.07	9.54	6.71	6.82	4.14	5.64	0.10	0.09
Val145	11.26	12.18	9.19	9.18	1.00	2.79	0.08	0.02
Ala146	9.65	10.57	7.62	7.65	0.68	3.05	0.02	0.01
Leu150	9.36	9.9	8.64	8.55	0.27	1.99	0.31	0.14

Table S5. Helical content of the amino acid sequence (Asp129 to Tyr140) within the CD loop of each leptin molecule in the leptin:LepR complexes with varying leptin binding stoichiometry. Note: The term "Missing loop" in any column under "leptin helical content" indicates that the CD loop was not fully resolved in the respective leptin-LepR assembly. Leptin 1, leptin 2, and leptin 3 refer to the leptin molecules designated with chain IDs A, C, and E in the assemblies with PDB IDs 8AVF and 8AVO, and with chain IDs D, E, and F in the assemblies with PDB IDs 8X80 and 8X81. Additionally, leptin 1 and leptin 2 refer to the leptin molecules designated with chain IDs C and D in the assembly with PDB ID 8DH9.

PDB ID	Binding stoichiometry of leptin:LepR	Helical content leptin 1	Helical content leptin 2	Helical content leptin 3
8AVF (2)	Closed 3:3	76.9%	76.9%	76.9%
8AVO (2)	Open 3:3	76.9%	76.9%	76.9%
8DH9 (10)	Open 2:2	92.3%	92.3%	
8X80 (11)	Open 3:3	Missing loop	84.6%	69.2%
8X81 (11)	Open 3:3	Missing loop	84.6%	100%

Table S6. Statistical evaluation of the distribution curves presented in Fig. S6 in the main text. The WT/P64S mean value represents the calculated mean distance or RMSD in Ångström from the geometric center distance or RMSD probability distribution curve. The WT/P64S initial value denotes the geometric center distance measured in the refined 3:3 leptin-LepR assembly. The WT/P64S variance indicates the variance of the sampled distance or RMSD value from the corresponding WT and P64S leptin-LepR complexes' simulations.

	WT mean value (Å)	P64S mean value (Å)	WT initial value (Å)	P64S initial value (Å)	WT variance (Å^2)	P64S variance (Å^2)
Distance leptin-CRH2	7.69	7.65	7.49	7.49	0.12	0.09
Distance leptin-IgD	8.16	7.94	7.93	7.93	5.78	0.22
RMSD leptin- CRH2	1.66	1.74			0.08	0.07
RMSD leptin-IgD	1.9	2.0			1.14	0.3

3. SUPPLEMENTAL RESULTS

Structural refinement of the leptin-LepR assembly

Given the low resolution (6.45 Å) of the 3:3 human leptin-LepR structure (PDB ID: 8AVF) (2), an initial 100 ns ATMD simulation was conducted to refine the complex structure. The pairwise interactions between the FNIII domains of each LepR ectodomain might be crucial for the receptor activation (2). Therefore, the volume of the core enclosed by these FNIII domains was monitored throughout the simulation trajectory to assess the stability of these interactions (Fig. S10). The evolution of the domains' core volume, as depicted in Fig. S10, exhibits a clear trend of fluctuation, with the volume oscillating around ~205,000 Å³ and concluding at a value similar to the initial volume. Visual inspection of the complex structures at various time points during the simulation indicates that the interactions between the FNIII domains were maintained throughout the simulation, with no signs of dissociation. Thus, the observed volume fluctuations appear to be normal variations of each FNIII domain relative to the others.

Within the closed 3:3 leptin-LepR complex, each leptin molecule interacts with two separate LepRs. Specifically, leptin utilizes its binding site II to engage the CRH2 domain of one LepR and its binding site III to interact with the IgD domain of another LepR (Fig. 1, *D* and *E*). For each monomeric leptin-LepR CRH2 or leptin-IgD structure in the initial cryo-EM structure, the amino acids at the interface between the two molecules were identified. Afterwards, their geometric center distances were monitored throughout the simulation (Fig. S11, *A* and *C*). Within the cryo-EM structure, the interfacial distance of the leptin-LepR CRH2 and leptin-LepR IgD are approximately 8.0 Å and 7.6 Å, respectively (Fig. S4). Given the high affinity of the CRH2 domain for leptin, it is unsurprising that the geometric

distances for all the leptin-CRH2 complexes remained stable at ~8.0 Å throughout the simulation (Fig. S11 *A*). The RMSD evolution of the complexes relative to the initial cryo-EM structure (Fig. S11 *B*) also indicates a stable structural conformation, with RMSD values consistently around ~3.0 Å.

Regarding the interactions between leptin and the LepR IgD domain, the geometric center distances for the leptin2-LepR1 IgD and leptin3-LepR2 IgD complexes (red and blue curves in Fig. S11 *C*) remained approximately 8.0 Å throughout the simulation. The RMSD evolution for these two complexes (red and blue curves in Fig. S11 *D*) also remained stable at ~2.0 Å relative to the initial cryo-EM structure. In contrast, the leptin1-LepR3 IgD complex underwent more significant structural rearrangement, with the distance between the binding interfaces expanding from less than 8.0 Å at the start of the simulation and fluctuating around 10.0 Å (green curve in Fig. S11 *C*). The RMSD evolution of the leptin1-LepR3 complex (green curve in Fig. S11 *D*) originally showed an increase and then fluctuated around ~3.0 Å until the end of the simulation.

The leptin's AB loop (from Thr48 to Thr71) has been proposed to adopt a flexible conformation (12,13), but gains order when bound to LepR (7,10,13,14,15). Compared to the unbound leptin, the AB loop is significantly less flexible in leptin2 and leptin3 when interacting with the LepR1 and LepR2 IgDs, respectively (Fig. S12, *B* and *C*), corroborating the previous findings. Although the AB loop of leptin1 when bound to the LepR3 is still less flexible than that of the unbound leptin, it is more flexible compared to leptin2 and leptin3. Together with the distance and RMSD data (Fig. S11), this suggests weaker interactions between leptin1 and the IgD domain compared to the other two leptin molecules. As Saxton et al. (2023) demonstrated that the recruitment of two LepR molecules is already sufficient for full receptor activation, a 3:3 leptin-LepR complex with one weak leptin-LepR IgD interaction is unlikely to impair the receptor signal transduction capacity (10).

From the refinement simulation, it is evident that the interactions between each leptin molecule and its high-affinity CRH2 domain within the closed 3:3 leptin-LepR complex were stably maintained throughout the simulation. Additionally, the pairwise interactions between the FNIII domains remained stable. Although one leptin molecule showed a tendency to dissociate from its weak-affinity binding site, the IgD, this should not impact the complex's signaling capacity. Finally, the complex structure extracted at the 100 ns time point was used as the input structure for subsequent simulations of the WT and MT.

4. REFERENCES

1. Robert, X., & Gouet, P. (2014). Deciphering key features in protein structures with the new ENDscript server. Nucleic Acids Research, 42(W1), W320-W324.

2. Tsirigotaki, A., Dansercoer, A., Verschueren, K. H. G., Marković, I., Pollmann, C., Hafer, M., Felix, J., Birck, C., van Putte, W., & Catteeuw, D. (2023). Mechanism of receptor assembly via the pleiotropic adipokine Leptin. Nature Structural & Molecular Biology, 30(4), 551-563.

3. Funcke, J.-B., Moepps, B., Roos, J., von Schnurbein, J., Verstraete, K., Fröhlich-Reiterer, E., Kohlsdorf, K., Nunziata, A., Brandt, S., & Tsirigotaki, A. (2023). Rare antagonistic leptin variants and severe, early-onset obesity. New England Journal of Medicine, 388(24), 2253-2261.

4. von Schnurbein, J., Zorn, S., Nunziata, A., Brandt, S., Moepps, B., Funcke, J.-B., Hussain, K., Farooqi, I. S., Fischer-Posovszky, P., & Wabitsch, M. (2024). Classification of Congenital Leptin Deficiency. The Journal of Clinical Endocrinology & Metabolism, dgae149.

5. Dominguez, C., Boelens, R., & Bonvin, A. M. J. J. (2003). HADDOCK: a protein− protein docking approach based on biochemical or biophysical information. Journal of the American Chemical Society, 125(7), 1731–1737.

6. Honorato, R. v., Trellet, M. E., Jiménez-García, B., Schaarschmidt, J. J., Giulini, M., Reys, V., Koukos, P. I., Rodrigues, J. P., Karaca, E., & van Zundert, G. C. P. (2024). The HADDOCK2. 4 web server for integrative modeling of biomolecular complexes. Nature Protocols, 19(11), 3219-3241.

7. Carpenter, B., Hemsworth, G. R., Wu, Z., Maamra, M., Strasburger, C. J., Ross, R. J., & Artymiuk, P. J. (2012). Structure of the human obesity receptor leptin-binding domain reveals the mechanism of leptin antagonism by a monoclonal antibody. Structure, 20(3), 487-497.

8. Vangone, A., Rodrigues, J., Xue, L. C., van Zundert, G. C. P., Geng, C., Kurkcuoglu, Z., Nellen, M., Narasimhan, S., Karaca, E., & van Dijk, M. (2017). Sense and simplicity in HADDOCK scoring: Lessons from CASP-CAPRI round 1. Proteins: Structure, Function, and Bioinformatics, 85(3), 417-423.

9. Ngo, Q. B., & Juffer, A. H. (2024). Theoretical Investigations of a point mutation affecting H5 Hemagglutinin's receptor binding preference. Computational Biology and Chemistry, 108189.

10. Saxton, R. A., Caveney, N. A., Moya-Garzon, M. D., Householder, K. D., Rodriguez, G. E., Burdsall, K. A., Long, J. Z., & Garcia, K. C. (2023). Structural insights into the mechanism of leptin receptor activation. Nature Communications, 14(1), 1797.

11. Xie, Y., Li, X., Qi, J., Shang, G., Lu, D., & Gao, G. F. (2023). Structural plasticity of human leptin binding to its receptor LepR. HLife, 1(2), 115-123.

12. Danielsson, J., Noel, J. K., Simien, J. M., Duggan, B. M., Oliveberg, M., Onuchic, J. N., Jennings, P. A., & Haglund, E. (2020). The pierced lasso topology leptin has a bolt on dynamic domain composed by the disordered loops I and III. Journal of Molecular Biology, 432(9), 3050-3063.

13. Funcke, J.-B., Moepps, B., Roos, J., von Schnurbein, J., Verstraete, K., Fröhlich-Reiterer, E., Kohlsdorf, K., Nunziata, A., Brandt, S., & Tsirigotaki, A. (2023). Rare antagonistic leptin variants and severe, early-onset obesity. New England Journal of Medicine, 388(24), 2253-2261.

14. Mancour, L. v, Daghestani, H. N., Dutta, S., Westfield, G. H., Schilling, J., Oleskie, A. N., Herbstman, J. F., Chou, S. Z., & Skiniotis, G. (2012). Ligand-induced architecture of the leptin receptor signaling complex. Molecular Cell, 48(4), 655-661.

15. Moharana, K., Zabeau, L., Peelman, F., Ringler, P., Stahlberg, H., Tavernier, J., & Savvides, S. N. (2014). Structural and mechanistic paradigm of leptin receptor activation revealed by complexes with wild-type and antagonist leptins. Structure, 22(6), 866-877.
